# Prion Protein and Shadoo Are Involved in Overlapping Embryonic Pathways and Trophoblastic Development

**DOI:** 10.1371/journal.pone.0041959

**Published:** 2012-07-30

**Authors:** Bruno Passet, Rachel Young, Samira Makhzami, Marthe Vilotte, Florence Jaffrezic, Sophie Halliez, Stéphan Bouet, Sylvain Marthey, Manal Khalifé, Colette Kanellopoulos-Langevin, Vincent Béringue, Fabienne Le Provost, Hubert Laude, Jean-Luc Vilotte

**Affiliations:** 1 INRA, UMR1313, Génétique Animale et Biologie Intégrative, INRA, Jouy-en-Josas, France; 2 INRA, UR892, Virologie et Immunologie Moléculaires, INRA, Jouy-en-Josas, France; 3 Laboratory of Inflammation, Gestation and Autoimmunity, J. Monod Institute, UMR 7592 (CNRS and University Diderot), Paris, France; University of Leicester, United Kingdom

## Abstract

The potential requirement of either the Prion or Shadoo protein for early mouse embryogenesis was recently suggested. However, the current data did not allow to precise the developmental process that was affected in the absence of both proteins and that led to the observed early lethal phenotype. In the present study, using various *Prnp* transgenic mouse lines and lentiviral vectors expressing shRNAs that target the Shadoo-encoding mRNA, we further demonstrate the specific requirement of at least one of these two PrP-related proteins at early developmental stages. Histological analysis reveals developmental defect of the ectoplacental cone and important hemorrhage surrounding the *Prnp*-knockout-*Sprn*-knockdown E7.5 embryos. By restricting the RNA interference to the trophoblastic cell lineages, the observed lethal phenotype could be attributed to the sole role of these proteins in this trophectoderm-derived compartment. RNAseq analysis performed on early embryos of various *Prnp* and *Sprn* genotypes indicated that the simultaneous down-regulation of these two proteins affects cell-adhesion and inflammatory pathways as well as the expression of ectoplacental-specific genes. Overall, our data provide biological clues in favor of a crucial and complementary embryonic role of the prion protein family in *Eutherians* and emphasizes the need to further evaluate its implication in normal and pathological human placenta biology.

## Introduction

The Prion protein, PrP, is the best known member of the prion protein family due to its pivotal role in transmissible spongiform encephalopathies [1–3 for reviews]. However, the physiological function of this ubiquitously expressed protein is still unclear, and the same is largely true for the related Shadoo and Doppel proteins. Various roles in neuroprotection, cellular homeostasis, response to oxidative stress, cell proliferation and differentiation, synaptic function and signal transduction have been proposed for PrP [4]–[7]. Shadoo was recently shown to possess neuro- and stress-protective properties [8]–[10] whereas inactivation of the Doppel-encoding gene in mice resulted in male infertility associated with strain-related variable sperm maturation defects [11], [12]. The difficulty to define a precise role for PrP partially comes from the observation that PrP-encoding gene-knockout (*Prnp* ^KO^) mice [13], [14], cattle [15] and goat [16] suffer from no drastic developmental phenotype. It was hypothesized that another host-encoded protein is able to compensate for the lack of PrP [17]. Shadoo, which shares some spatial regulation and properties with PrP, appears to be a good candidate for being this hypothetical, host-encoded PrP-like protein [18].

The developmental regulation of the mouse *Prnp* gene suggested a possible involvement of PrP in embryogenesis [19]–[22]. The two other prion-related proteins are also expressed in early developmental stages according to the available EST databases and to recent reports [23], [24]. The hypothesis of an embryonic role of the PrP protein family was recently reinforced by the observation that PrP and Shadoo are required for early mouse embryogenesis, as lethality was observed around E10.5 in *Sprn*-(Shadoo-encoding gene-)knockdown, *Prnp*-knockout embryos [25]. These data also suggested that the physiological role of these proteins may have to be investigated at early developmental stages.

The aim of this study was to get further insight into the biological function of the prion protein family during early embryogenesis using transcriptomic analysis and cell lineage-specific gene targeting.

## Results

### Shadoo-knockdown (Sprn^KD^)-induced Embryonic Lethality Requires the Lack of PrP Proteins

We have previously reported that the injections of two independent sh-RNA lentiviral solutions (LSI : SIGMA TRCN0000179960 and LS2 : SIGMA TRCN0000184740), both targeting the mouse *Sprn* transcript, induce embryonic lethality in FVB/N *Prnp* ^KO^ embryos that was not detected on an FVB/N genetic background [25]. However, since then, evidence has been published highlighting other differences than *Prnp* between these two genetic backgrounds. *Prnp*-physically linked 129-derived loci were reported to be conserved in FVB/N *Prnp* ^KO^ mice alongside the *Prnp* locus itself [26]. Although unlikely, such non-*Prnp* loci could be involved in the observed lethality associated with *Sprn* knockdown in FVB/N *Prnp* ^KO^ embryos. RNAi is known to potentially induce off-target effects ([27] for recent review), and such off-target events might interfere with the expression of some of the 129-derived genes indirectly selected during the *Prnp* knockout experiment. Alternatively, alleles of these genes might differentially modulate specific pathways that are involved during the knockdown process, leading to a lethal phenotype. To assess how specific the previously described phenotype is to the double knockout/knockdown and the potential involvement of additional genes coming along with the *Prnp* knockout allele, we took advantage of our recent derivation, following micro-injection of a transgene based on the phgPrP-vector in FVB/N *Prnp* ^KO^ eggs, of a homozygous P10 transgenic mouse line that expresses the ovine PrP protein at physiological levels. In this transgenic line, all the 129-additional genes of the FVB/N *Prnp* ^KO^ mice are present alongside a functional ovine *Prnp* transgene. Injection of the sh-RNA lentiviral solution targeting FoxL2 in P10 mice gave results statistically similar to those previously observed for FVB/N or FVB/N *Prnp* ^KO^ eggs (Table 1 and [25]), arguing that the P10 genetic background is not associated with an unusual susceptibility to lentiviral infection and/or RNA interference. Injection in P10 mouse eggs of the shRNA targeting *Sprn* lentiviral solution (LS2 in [25]) resulted in an embryonic survival rate similar to that observed in FVB/N and significantly higher from that previously detected for FVB/N *Prnp* ^KO^ mice (Table 1, p<0.05). The induced LS2 lethality is thus dependent on the presence or not of a functional PrP-encoding gene and not on other 129 *Prnp* ^KO^-associated loci.

**Table 1:** Effect of ShRNA-mediated *Sprn* knockdown on embryo resorption at E11.5.

Lentivirus	FoxL2	FoxL2	FoxL2	Shadoo (LS2)	Shadoo (LS2)	Shadoo (LS2)	None
Genetic Background	FVB/N	FVB/N *Prnp* ^0/0^	P10	FVB/N	FVB/N *Prnp* ^0/0^	P10	P10
Implanted	56	41	79	101	128	105	28
Resorbed	28	16	39	49	96	36	5
% Resorbed	50	39	49	48.5	75*	34.3	17.8

* p<0.05 (x^2^ test) when compared to any of the FoxL2 results and to LS2 on P10 and FVB/N genetic backgrounds.

These data are a compilation of at least 2 independent experiments. No statistically significant variability was observed between the analyzed litters (3 for P10, more than 4 for the other lentiviral infections).

### Histological Analysis of FVB/N Prnp^KO^ Sprn^KD^ Embryos Reveals Ectoplacental Cone Defects and Local Hemorrhage

**Figure 1:**
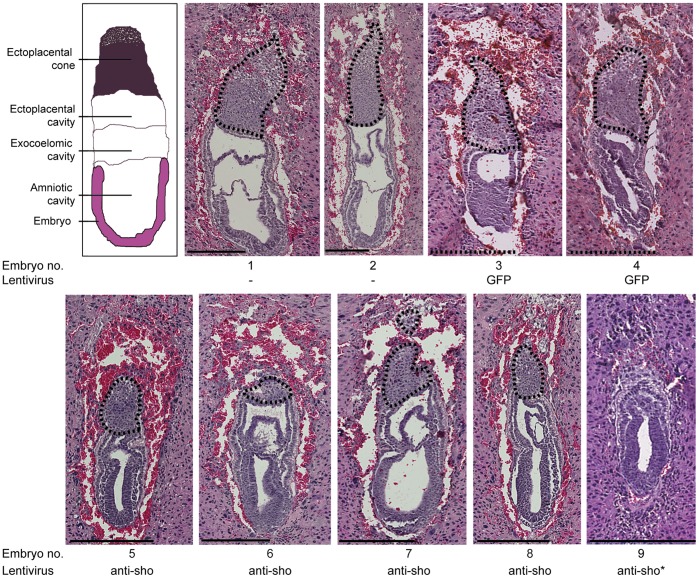
Histological analysis of E7.5 embryos. E7.5 embryos were fixed and stained by hematoxylin, eosin, and saffron. Top left: schematic representation of a mouse E7.5 embryo. 1,2: FVB/N *Prnp^KO^* embryos. 3,4: FG12-injected FVB/N *Prnp^KO^* embryos. 5–8: LS2-injected FVB/N *Prnp^KO^* embryos. 3–8: embryos that were injected at the zygotic stage. 9: LS2-infected FVB/N *Prnp^KO^* embryos. *: infection performed at the blastocyst stage. Interesting features include i) the size differences between injected and non-injected embryos, ii) the relatively important hemorrhagic tissue that is totally surrounding the LS2-injected FVB/N *Prnp^KO^* embryos 5, 6 and 8, iii) the developmental defect of the ectoplacental cone (area surrounded using a dashed line ) of all the LS2-injected FVB/N *Prnp^KO^* embryos (5–8) that even leads to its nearly complete disappearance in embryo 6 and iv) the important developmental delay and the total disorganization of the extra-embryonic ectoderm and of the ectoplacental cone of embryo 9. Scale: 250 µm. Sho: *Sprn*.

Comparative histological analyses of E7.5 embryos were performed between FVB/N *Prnp* ^KO^ and FVB/N *Prnp* ^KO^ embryos injected at the zygotic stage with either a FG12 lentiviral solution, used as a control as it only encodes GFP (http://www.addgene.org/14884) and could thus allow tthe control of the lineage specific lentiviral infection (see below), or an shRNA targeting *Sprn* LS2-lentiviral solution [25]. This developmental stage was chosen as a compromise between the early embryonic lethality observed following LS2 injection ([25] and current data) and a developmental timing that could allow the assessment of some embryonic lineage differentiation. FG12-injected embryos (Figure 1, #3 and 4) were found to be slightly developmentally delayed as compared to non-injected embryos (Figure 1, #1 and 2), and some were surrounded by minor hemorrhage, attested by pinkish-red stained red blood cells (#3 for example, 2 out of the 6 analyzed embryos), but otherwise they did not present other major defects (6/6 embryos). LS2-injected embryos were similarly developmentally delayed (7/7 embryos), suggesting that this phenotype is associated with the *in vitro* manipulation of the eggs, but they were fully comparable in size and developmental stage to FG12-injected controls. Most importantly, in contrast to control embryos (Figure 1), LS2-injected embryos were characterized by reduced ectoplacental cone surfaces. Compared to control embryos, their cones were disorganized, with a notably reduced and even fragmented invasive chorionic trophoblast cell layer (Figure 1, #7 and 8). Such a phenotype was never observed in the above-mentioned control embryos (13/13). In addition, all the seven analyzed embryos were fully surrounded by large hemorrhagic lacunae containing lots of red blood cells (Figure 1, #5 to 8).

### Trophoblastic-restricted RNA Interference Induces Embryonic Lethality

The embryonic lethality reported for FVB/N *Prnp* ^KO^ *Sprn* ^KD^ embryos occurred at a developmental stage at which trophoblastic lineage cells proliferate and differentiate to provide adequate nutriments and metabolic exchanges to the fetus via a developing, expanding and maturing placenta. Histological analysis of the embryos clearly pointed to a defect in the ectoplacental cone development (Figure 1). The ectoplacental cone derives from the outer trophectoderm. Separation of the trophectoderm from the inner cell mass is the first lineage distinction that occurs during mammalian development at the blastocyst stage. To assess whether the lethal phenotype could be associated with a developmental defect restricted to the embryo or to trophectoderm-derived, extra-embryonic tissues, we took advantage of the capacity of lentiviral vectors to specifically infect the trophoblast when used on blastocysts ([28], [29], Figure S1).

The obtained results showed no statistical difference in the percentage of surviving embryos when lentiviral solutions of ShRNA-targeting FoxL2 on either genetic background or of ShRNA LS2 targeting *Sprn* on FVB/N embryos were used (Table 2). These results highlighted that i) this targeted lentivirus delivery protocol leads to identical survival rate compared to zygotic injection when a gene known to have no obvious function in the placental development such as FoxL2 is downregulated and ii) that *Srpn* downregulation does not appear to interfere with the placenta development of FVB/N embryos or at least with no detectable incidence on the survival rate. The ShRNA-targeting *Sprn* LS2 lentiviral solution applied on FVB/N *Prnp* ^KO^ blastocysts led to a strikingly different output with a statistically higher resorption rate (100% versus <50%, Table 2, p<0.05). A similar trend was observed when the ShRNA-targeting *Sprn* LS1 lentiviral solution was used to infect FVB/N *Prnp* ^KO^ blastocysts, resulting in a resorption rate above 60% as compared with less than 40% with the ShRNA-targeting FoxL2 lentiviral solution (Table 2). The lower rate observed with LS1 is consistent with the previously reported overall lower capacity of this lentiviral solution to induce a phenotype as compared with LS2 [25] likely to be in relation with its apparent lower efficiency to knockdown *Sprn* (see Fig. 1 in [25]). Overall, these results were not statistically different from those for zygotic lentiviral infections (Tables 1 and 2).

**Table 2:** Effect of trophoblastic-restricted ShRNA- mediated *Sprn* knockdown on embryo resorption at E13.5.

Lentivirus	FoxL2	FoxL2	Shadoo (LS2)	Shadoo (LS2)	Shadoo (LS1)
**Genetic Background**	FVB/N	FVB/N *Prnp* ^0/0^	FVB/N	FVB/N *Prnp* ^0/0^	FVB/N *Prnp* ^0/0^
**Implanted**	37	24	44	40	66
**Resorbed**	19	9	17	40	40
**% Resorbed**	51.3	37.5	38.6	100*	60.6

* p<0.05 (x^2^ test) when compared to any of the other results.

These data are a compilation of at least 2 independent experiments. No statistically significant variability was observed between the analyzed litters (more than 4 for each lentiviral infections).

Histological analyses performed on E7.5 FVB/N *Prnp* ^KO^ embryos that had been infected at the blastocyst stage with either lentiviral LS2 or FG12 solutions revealed that while FG12 did not interfere with the developmental process of the embryo (data not shown), the use of LS2 resulted in i) an overall developmental delay associated with no other obvious major defects and ii) a disorganization of the extra-embryonic ectoderm and ectoplacental cone, independently of the observed retarded developmental stage with a nearly complete disappearance of the invasive chorionic trophoblast cell layer (Figure 1, #9). This phenotype is thus similar, although more pronounced, to that observed after zygotic infection. It should be mentioned that these embryos were not surrounded by significant hemorrhage, suggesting that this phenotype is either associated with the zygotic stage of injection or, most probably, not yet apparent due to the developmental delay and the much reduced decidualisation. Altogether, these observations indicate that a dysfunction of the trophoblastic lineage induced by the knockdown of *Sprn* in the absence of PrP is sufficient to induce a rate of embryonic lethality similar to the one associated with the *Sprn* ^KD^ *Prnp* ^KO^ genetic background.

### Analysis of Sprn^KD^ Embryos Reveals Differential Expression of Few Genes Involved in Major Developmental Processes along Prnp

RNASeq analysis was performed on pools of E6.5 and E7.5 FVB/N and FVB/N *Prnp* ^KO^ embryos that were injected at the pronuclear stage with either LS1 or LS2 ShRNA targeting *Sprn* lentiviral solutions, as previously described [25]. These two developmental time-points were chosen according to the lethality observed in FVB/N *Prnp* ^KO^ *Sprn* ^KD^ embryos that was found to be already substantial at E8.5, refining the timescale previously described [25]. Genes were considered to be differentially expressed when their deregulation was observed for both LS1 and LS2. Genes that were deregulated by only one of these ShRNAs were considered to result either from RNA-interference off-target effect or from variation between biological replicates. Of note is the observation that all the differentially expressed genes were similarly either upregulated or downregulated by both ShRNA when compared to FVB/N wild-type embryos (Table S1).

Validation by PCR analysis of RNASeq data obtained during this experiment has been previously reported [30]. *Sprn* itself was not detected as a differentially expressed gene due to the too low number of reads for its transcript (<5 reads). This quantitative approach confirmed that this locus is only expressed at low levels in early embryos, at least 100-fold less than *Prnp*. However, assessment of the *Sprn*-down-regulation through LS2-induced RNA interference had already been reported [25] and confirmed in the present experiment by RT-PCR and QPCR, with an overall >65% level of inhibition (Figure S2).

**Figure 2:**
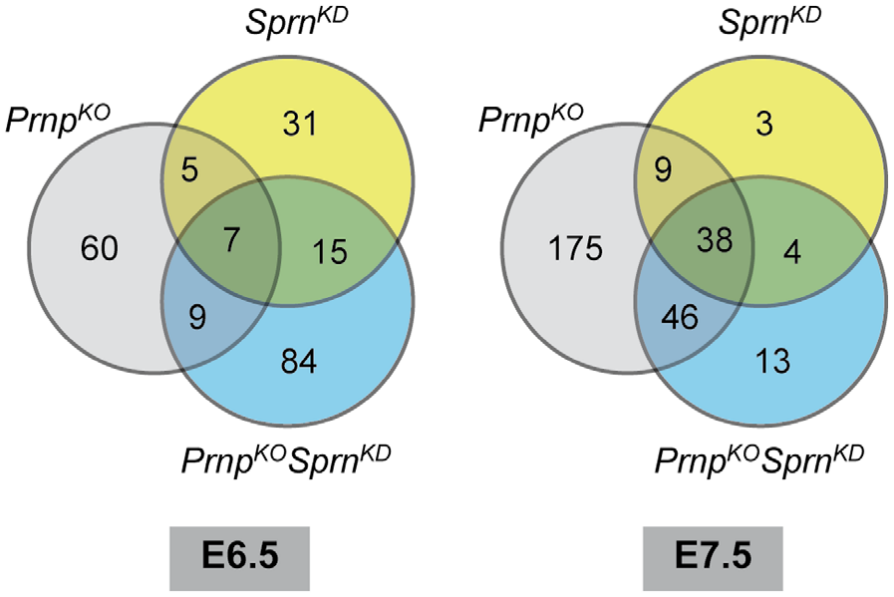
Venn diagram of the number of differentially expressed genes. A venn diagram of the number of genes differentially expressed between *Prnp^KO^*, *Sprn* ^KD^, *Prnp* ^KO^ *Sprn* ^KD^ embryos and their wild type counterparts is given at E6.5 and E7.5.

*Sprn*-downregulation on a FVB/N genetic background induces the differential expression of 58 and 54 transcripts at E6.5 and E7.5, respectively, of which, strikingly, only 5 were found at both developmental stages (Figure 2 and Table S1). This total represents less than 0.14% of all the identified transcripts. Of note is the deregulation in *Sprn^KD^* embryos of genes specifically expressed in the ectoplacental cone, such as the prolactin-related Prl2C5, Prl2a1 [31]–[34], and/or in inflammatory response, such as interleukin 15 (IL15), complement factor H, granzymes and transmembrane serine protease members, Prss28 and 29 [35], in good correlation with the histological phenotype. Whereas at E6.5, 12 (22%) of the differentially expressed genes were also differentially expressed in *Prnp* ^KO^ embryos ([30] and Figure 2), at E7.5, 47 (87%) of the genes differentially expressed in *Sprn^KD^* embryos were also differentially expressed in E7.5 *Prnp* ^KO^ embryos ([30] and Figure 2).

The same analysis performed on FVB/N *Prnp* ^KO^ genetic background reveals the differential expression of 115 and 101 genes at E6.5 and 7.5, respectively when compared to FVB/N embryos, of which only two were found at both developmental stages (Figure 2 and Table S1). At E6.5, 84 genes (73%) were found to be specifically deregulated in *Sprn* ^KD^ *Prnp* ^KO^ embryos compared to their *Prnp* ^KO^ or *Srpn* ^KD^ counterparts whereas this number drops to 13 (13%) at E7.5. This observation highlights that at early developmental stages, the simultaneous down-regulation of these two genes appears to impact specific biochemical pathways that are not disturbed or to a much lesser extent in embryos invalidated for only one of them. Within these affected genes, several were also specifically expressed in the ectoplacental cone, such as the prolactin-related Prl2C1 and Prl2C3 loci.

*In silico* analysis of the observed transcriptomic alterations identified few pathways that encompass more than 10 of the differentially expressed genes (Table 3). The top functions associated with these pathways, molecular transport, hematological system development, cardiovascular system development, skeletal and muscular system developments, hair and skin developments, were identical to those recently reported for similar transcriptomic analysis of FVB/N *Prnp* ^KO^ embryos [30] and/or for PrP function in adult tissues [4]–[7]. Interestingly, the above-mentioned genes that were specifically deregulated in *Sprn* ^KD^ *Prnp* ^KO^ E6.5 embryos were identified as being involved in functions such as developmental and genetic disorders, cellular movement and development (Table 3 and Table S1). Many of these genes encode for proteins involved in the extracellular matrix, such as collagens, its remodeling, such as metalloproteinases and the biglycan (*Bgn*) locus, and cell-cell interactions such as cadherins. Since trophectoderm is the first differentiated tissue to form with cells needing adhesive structures, this observation is in accordance with the histological observed defect of the ectoplacental cone development.

**Table 3:** List of top-function clusters for differentially expressed genes in *Sprn*-knockdown embryos.

Developmental Stage/Genetic Background	Top Functions	Genes
**E6.5 FVB/N ** ***Sprn*** **^KD^ versus WT**	Cell Signaling. Cellular Growth and Differentiation. Hematological System	**ABCA1**, **ADAMTS1,** **ANGPT2**, CDH22, **CIDEA**, **CTSK, CXCL13,** **DIO2**, **IL15, INHBA,** **LMCD1**, PCSK5, **TIMP3**, **TNNC1**, TPSAB1
	Developmental and Genetic Disorder	RMRP, RPPH1
**E6.5 FVB/N ** ***Prnp*** **^KO^** ***Sprn*** **^KD^ versus WT**	Developmental and Genetic Disorder, Metabolic Disease	**ADH1C, C3, C1QB, CD74, CDH11, CFH, COL1A1, COL1A2, CTSK, HLA-DRB1, IL15, LGALS3BP, MMP2, MMP238, RHOJ, SERPINA1**
	Lipid metabolism, Molecular transport	**CCDC80,CDH11, CHODL, EAF2, GJA4, ITIH5, KIAA1324, SDCBP2, SEMA5A, SULT1A1, TCF23, TMEM204, TMEM45A, WFDC2**
	Cell cycle, Reproductive system development and function, cellular development	**AGR2, COL1A1, CWH43, GGH, HTRA3, ITGA8,** Prl2a1, Prl2c2, **RNASE1, ROBO2, SPACA7**
	Cellular movement, Hematological system development and function	**ACTG2, CXCL14, DIO2, EDNRB, MYLK, NUPR1, PAX8, PCP4, PENK, WNT5A**
**E6.5 FVB/N ** ***Prnp*** **^KO^** ***Sprn*** **^KD^ versus ** ***Prnp*** **^KO^**	Tissue Morphology. Cellular Growth and Differentiation. Hematological System	**ACTA2, ACTG2, AGR2, CNN1, CTSK, IL15**, MIXL1, **MYH11, MYLK, NUPR1, SAA3P, TAGLN**
	Genetic Disorder. Hematological Disease	**PCP4**, PFAS, **SYNPO2**, TMEM102
	Developmental and Genetic Disorder	**RMRP**, **RPPH1**
**E7.5 FVB/N ** ***Sprn*** **^KD^ versus WT**	Cell Cycle. Cardiovascular System Development and Function, Organismal Development	A2M, ANGPT2, ARG1, DCN, DIO3, GJA1, LYVE1, **MMP7**, PRRX2, PTN, S100A4, SLC2A12, SLPI, **TDGF1**, TNFRSF11B
	Cellular Development. Cellular Growth and Proliferation. Hair and Skin Development	ANXA8, COL5A2, CYP11B1, FBLN2, HAVCR2, MFAP5, **NAPSA**, PDZK1IP1, **PRAP1**, SMOC2
	Lipid Metabolism. Small Molecule Biochemistry. Carbohydrate Metabolism	CRIP1, EMCN, HSPB7, PTRF, TDO2
**E7.5 FVB/N ** ***Prnp*** **^KO^** ***Sprn*** **^KD^ versus WT**	Connective tissue development and function, skeletal and muscular system development	ANGPT2, CRYAA, CYP286, DES, DIO3, FBLN2, FBN1, H5D11B1, LBP, LYVE1, MFAP5, PTGS1, PTN, SRD5A1, **TDGF1**, TNFRSF11B
	Cellular development, Embryonic development	ANXA8, COL5A2, CRIP1, CTSO, CYP286, FBLN2, HOXA11, IGSF11, Ly6a, **NDUFAF1**, OLFML3, PTRF, SDPR, TDO2, ZNF503
**E7.5 FVB/N ** ***Prnp*** **^KO^** ***Sprn*** **^KD^ versus ** ***Prnp*** **^KO^**	Embryonic Development	ALDH1A2, ARG1, CDX1

Bold faced genes: upregulated genes in *Sprn*-knockdown embryos. Un-bolded genes: downregulated genes in *Sprn*-knockdown embryos.

We also performed similar *in silico* analysis on genes that were differentially expressed between *Sprn* ^KD^ *Prnp* ^KO^ embryos and *Prnp* ^KO^ or *Sprn* ^KD^ embryos at both developmental stages (Table 3 and Table S1). At E6.5, three biological pathways were identified for genes differentially expressed between *Sprn* ^KD^ *Prnp* ^KO^ and *Prnp* ^KO^ embryos, cellular growth and differentiation, hematological system development and disease, developmental and genetic disorder. It corresponded to top functions already documented. In E7.5 FVB/N *Prnp* ^KO^ *Sprn* ^KD^ embryos, the Ingenuity-detected pathway was suggestive of a general distress (Table 3), in agreement with the embryonic lethality that is observed in such animals [25]. Only few genes were found to be differentially expressed between *Sprn* ^KD^ *Prnp* ^KO^ and *Sprn* ^KD^ embryos, 8 at E6.5 and 14 at E7.5 (Table S1). Five out of the 8 identified genes at E6.5 (*Actg2, Myh11, Cnn1, Acta2* and *Pep4*) are involved in cellular movement and hematological system development and differentiation functions (Table 3). However, none of these genes were share between the two developmental stages and none were specifically deregulated in the *Sprn* ^KD^ *Prnp* ^KO^ embryos. At E7.5, 12 out of the 14 genes that were identified comparing *Sprn* ^KD^ *Prnp* ^KO^ and *Sprn* ^KD^ embryos were inversely differentially expressed by LS1 or LS2 It probably reflects the overall more pronounced phenotype observed with LS2-treated embryos.

## Discussion

While the inactivation of the sole *Prnp* gene did not affect the survival rate of mammals despite early embryonic expression [13]–[16], it induced embryonic lethality in Zebrafish [36], [37] arguing for the involvement of the Prion protein family in embryonic development. Such a lethal phenotype was also described in early FVB/N *Prnp* ^KO^ *Sprn* ^KD^ mouse embryos [25] suggesting that in mammals, the absence of *Prnp* was compensated by the expression of the PrP-related protein, Shadoo. Here we provide further evidence of the specificity of this compensation by showing that such a knockdown approach performed on a transgenic line recently established that expresses physiological levels of ovine PrP on an FVB/N *Prnp* ^KO^ genetic background, led to results similar to those obtained on FVB/N control mice.

The surviving FVB/N *Prnp* ^KO^ *Sprn* ^KD^ embryos suffer from a neural defect with a failure of closure of the cranial tube [25]. However, it is commonly accepted that such a default could not explain the observed lethality. As a major finding of this study, we show that trophoblastic-targeted *Sprn*-down-regulation led to developmental defects of the trophoblastic lineage in an FVB/N *Prnp* ^KO^ genetic background that were sufficient to explain the observed lethal phenotype. It does not preclude that other embryonic detrimental biological functions might be also affected. The above-mentioned absence of neural tube closure for example might be a direct consequence of a placental developmental defect [38], or directly result from embryonic transcriptomic alterations. It was indeed reported that alterations of trypsin-like serine protease activities, such as granzymes and PRSS found to be differentially expressed in *Prnp* ^KO^ *Sprn* ^KD^ embryos, could alter both the placental development and neurogenesis [39]. Although *Sprn* transcriptional regulation appears to be more tightly controlled than that of *Prnp* in terms of tissue-specificity [8], [21], [24], [40], we could detect *Sprn* expression in E10.5 extra-embryonic annexes, supporting the suggested role of these genes in the trophoblastic lineage (Figure S3 and [24]).

**Figure 3:**
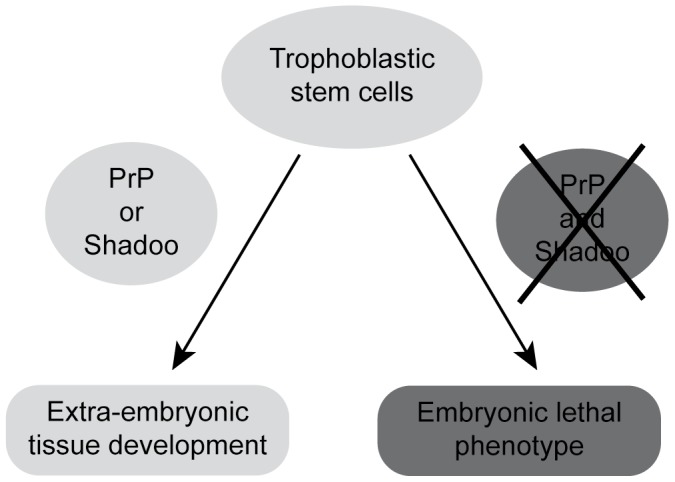
Schematic representation of the embryonic phenotype induced by the lack of PrP and Shadoo. The lethal consequence of the absence of PrP and Shadoo during early mouse embryogenesis is indicated.

Comparative analyses of E6.5 and E7.5 FVB/N *Prnp* ^KO^ or *Prnp* ^WT^ *Sprn* ^KD^ embryos with their wild-type FVB/N counterparts, performed by RNASeq, revealed that relatively few genes were differentially expressed following *Sprn* downregulation. Overall, these transcriptomic data suggested that Shadoo and PrP have complementary, not necessary overlapping, functions associated with cellular movement and hematological system development and differentiation. Such biological roles were already highlighted in *Prnp*-invalidated Zebrafish [37], [41] and mouse [30] embryos. It also emphasized Shadoo potential synergetic involvement in the development of extra-embryonic lineages with the differential expression of specific ectoplacental genes (Table 3). Strikingly few differentially genes were consistently found at both E6.5 and E7.5, suggesting a fast evolution/adaptation of the embryo biology at these early developmental stages. Furthermore, the histological and the pathways’ analyses at E6.5 and E7.5 suggest that the lack of expression of these two genes synergically affects the establishment of the chorioallantoic placenta (Figure 3). The trophectoderm-derived compartment is a differentiated tissue that forms with invading cells needing complex adhesive structures. Differential expression of several genes involved in the establishment, modeling and maintenance of the extracellular matrix as well as that of genes specifically expressed in the ectoplacental cone were observed only when both *Prnp* and *Sprn* genes were invalidated. It is likely to impair placental formation, expansion and maturation, thus reducing its invasive capacity and depriving the embryos of their vital resources. Consequently, it may result in the activation of macrophagic and pro-apoptotic reactions leading to embryonic resorption [36], [42]. Overall, our results suggest an as yet unknown function of the prion protein family in controlling the trophoblastic cell lineage maintenance and differentiation, potentially expending the involvement of these proteins in stem cell biology. It re-enforces the interest in looking at the prion protein family involvement in normal and pathological human placenta biology [43], [44].

During the revision process of this article, Daude et al. reported the creation and analysis of *Sprn*-knockout mice that did not produce embryonic lethality in combination with *Prnp*-invalidation, although the output of crosses between *Sprn* ^KO^ *Prnp* ^KO^ x *Sprn* ^KO^ *Prnp* ^KO^ mice was not apparently assessed (TableS1 in [45]). Three hypotheses were proposed by the authors that could conciliate their data with that of the previously published *Sprn*-knockdown ones [25] and of the present article; i) the use of similar but not identical genetic backgrounds, ii) an adaptation of the Sprn-knockout embryos to the lack of this protein and iii) an alteration of the *Sprn*-overlapping Mtg1 transcript expression level by the shRNA. This latter hypothesis can be ruled out by i) the location of the targeting sequences of LS1 and LS2 that are located outside the overlapping region between *Mtg1* and *Sprn* [25] and ii) the present analysis that indicates no alteration of the *Mtg1* gene expression by either LS1 or LS2 (Table S1 and data not shown). We are currently testing the former hypotheses by invalidation of *Sprn* in the FVB/N *Prnp*-knockout genetic background by the use of Zing Finger Nucleases.

## Materials and Methods

Animal experiments were carried out in strict accordance with the recommendations in the guidelines of the Code for Methods and Welfare Considerations in Behavioural Research with Animals (Directive 86/609EC). And all efforts were made to minimize suffering. Experiments were approved by the Local ethics committee of Jouy-en-Josas on the Ethics of Animal Experiments of the author’s institution, INRA (Permit Number RTA06-091). All animal manipulations were done according to the recommendations of the French Commission de Génie Génétique (Permit Number N°12931 (01.16.2003)).

### Generation of the P10 Transgenic Line

The ovine 136A154R171R PrP cDNA was inserted into the phgPrP half-genomic vector as previously described [46], leading to the P10 construct. The *Not*I-*Sal*I gel-purified, plasmid-free, insert was micro-injected into FVB/N *Prnp*-knockout (*Prnp* ^KO^) oocytes [13], [47]. A transgenic line, that expressed the transgene in its brain at physiological levels, could be derived and bred to a homozygous status.

### Lentiviral Injection in Mouse Embryos and Blastocysts

Intra-perivitellin space injections of lentiviral solutions were done according to [48]. Trophoblast-specific lentiviral infections were done as previously described [28], [29]. The sh-RNA lentiviral solutions used that target either mouse *Sprn* or mouse *FoxL2* were purchased from Sigma (LSI : TRCN0000179960, LSII : TRCN0000184740 and Lfox : TRCN0000086505) [25]. Chi2 statistical analyses of the differences observed for survival rates were performed.

### Histological Analysis

Collected embryos alongside their deciduas and uterine tissue were fixed in 4% PFA, dehydrated in ethanol before being embedded in paraffin and 5 µm sections cut on a microtome. Sections were stained by hematoxylin, eosin, and saffron then photographed using the Nanozoomer (Hamamatsu). On average, 50 sections per embryos were made and analyzed.

### RNAseq Analysis

Total RNA was isolated from pools of E6.5 and E7.5 mouse embryos. RNA extractions were performed using the RNeasy Lipid Tissue Mini kit (Qiagen cat # 75842). RNA concentration was calculated by electro-spectrophotometry and the RNA integrity checked with the Agilent Bioanalyser (Waldbroom, Germany).

RNA samples of 5 µg, obtained from around 30 embryos each collected from 3 to 4 females, were sent to GATC Biotech SARL for RNAseq analysis. A standard cDNA library was derived from each sample. These cDNAs were analyzed on an Illumina Genome Analyzer II with raw data output of up to 350 Mb and 42,000,000 reads per sample and a read length of 36 bases (single read). Sequence cleaning was done using Seqclean (http://compbio.dfci.harvard.edu/tgi/sofware/seqclean_README). Cleaned reads were mapped to the NCBI mouse transcript database (ftp://ftp.ncbi.nih.gov/genomed/M_musculus/RNA/) using BWA software [49].

Differentially expressed genes between FVB/N and FVB/N *Prnp* ^KO^ embryos were identified at 5% FDR using the DESeq package of the R software [50]. They were clustered using the software DAVID [51], then classified in pathways and networks by using Ingenuity (http://www.ingenuity.com/) and the GEPS application of Genomatix (http://www.genomatix.de).

## Supporting Information

Figure S1 **Trophoblastic-specific GFP expression pattern.** Trophoblastic-specific expression pattern of an ubiquitin-EGFP lentiviral-expressing vector (FG12, Addgene) was achieved in E10 mouse embryos following infection as described in Okada et al., 2007. No GFP signal was detected in non-infected control embryos of similar age. Images were merged using the AxioVision 4.8 software.
(TIF)

Figure S2 **Evidence for Sprn downregulation.** RT-PCR was performed on total RNA isolated from pooled E7.5 embryos. The used oligonucleotides and PCR conditions were as previously described (25). Actb: actin control RT-PCR. M: 1 kb ladder molecular weight marker (InVitrogen). 1: FVB/N embryos. 2: LS2-injected FVB/N embryos. 3: FVB/N *Prnp^KO^* embryos. 4: LS1-injected FVB/N *Prnp ^KO^* embryos.
(TIF)

Figure S3 **Evidence for** ***Sprn*** **expression in mouse placenta.** RT-PCRs were performed on total RNA isolated from E12 mouse placenta embryos (P1 and P2) and adult brain (Br). The used oligonucleotides and PCR conditions were as previously described (25). Actb: actin control RT-PCR. M: 1 kb ladder molecular weight marker (InVitrogen). + with reverse transcriptase. – without reverse transcriptase.
(TIF)

Table S1 **Complete list of genes differentially expressed in mouse embryos of various genotypes at E6.5 and E7.5.** The genotypes of the compared embryos are indicated in the top line. Upregulated genes are in green. Downregulated genes are in red. Indicated numbers in each case refer to the observed fold ratio (p Value). For data involving *Sprn* knockdown experiments, in each case the first set of numbers is that observed with LS1, the second set is that observed with LS2. ??: upregulation of the gene with no observed expression in the control genotype.
(DOC)
