# Supplementary figures and images for: Prion Protein and Shadoo Are Involved in Overlapping Embryonic Pathways and Trophoblastic Development
Source: PLoS One. 2012 Jul 30;7(7):e41959. doi: 10.1371/journal.pone.0041959 (PMC3408428; doi:10.1371/journal.pone.0041959)

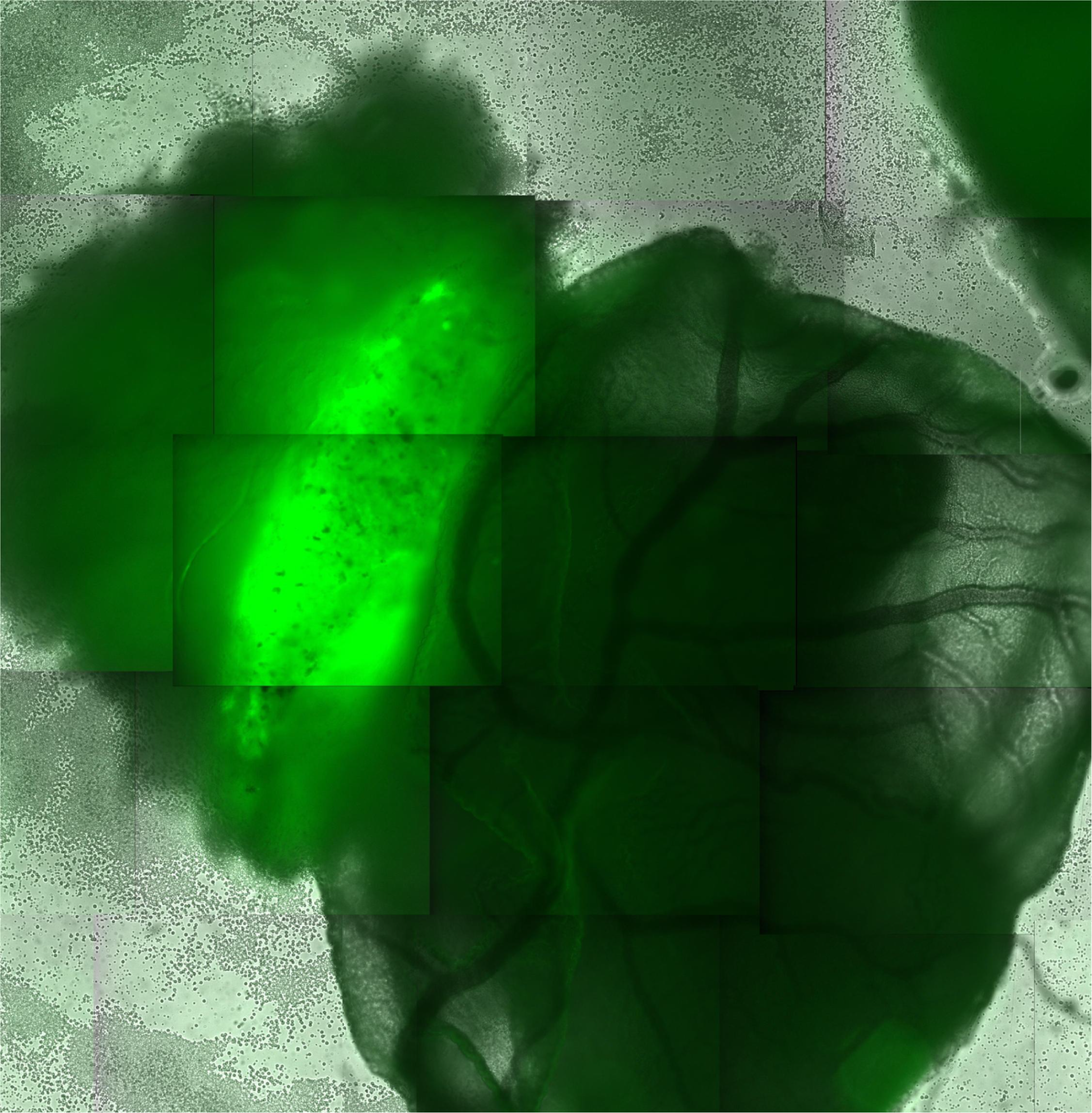

Supplement: Figure S1 — Trophoblastic-specific GFP expression pattern. Trophoblastic-specific expression pattern of an ubiquitin-EGFP lentiviral-expressing vector (FG12, Addgene) was achieved in E10 mouse embryos following infection as described in Okada et al., 2007. No GFP signal was detected in non-infected control embryos of similar age. Images were merged using the AxioVision 4.8 software. (TIF) [file pone.0041959.s001.tif]

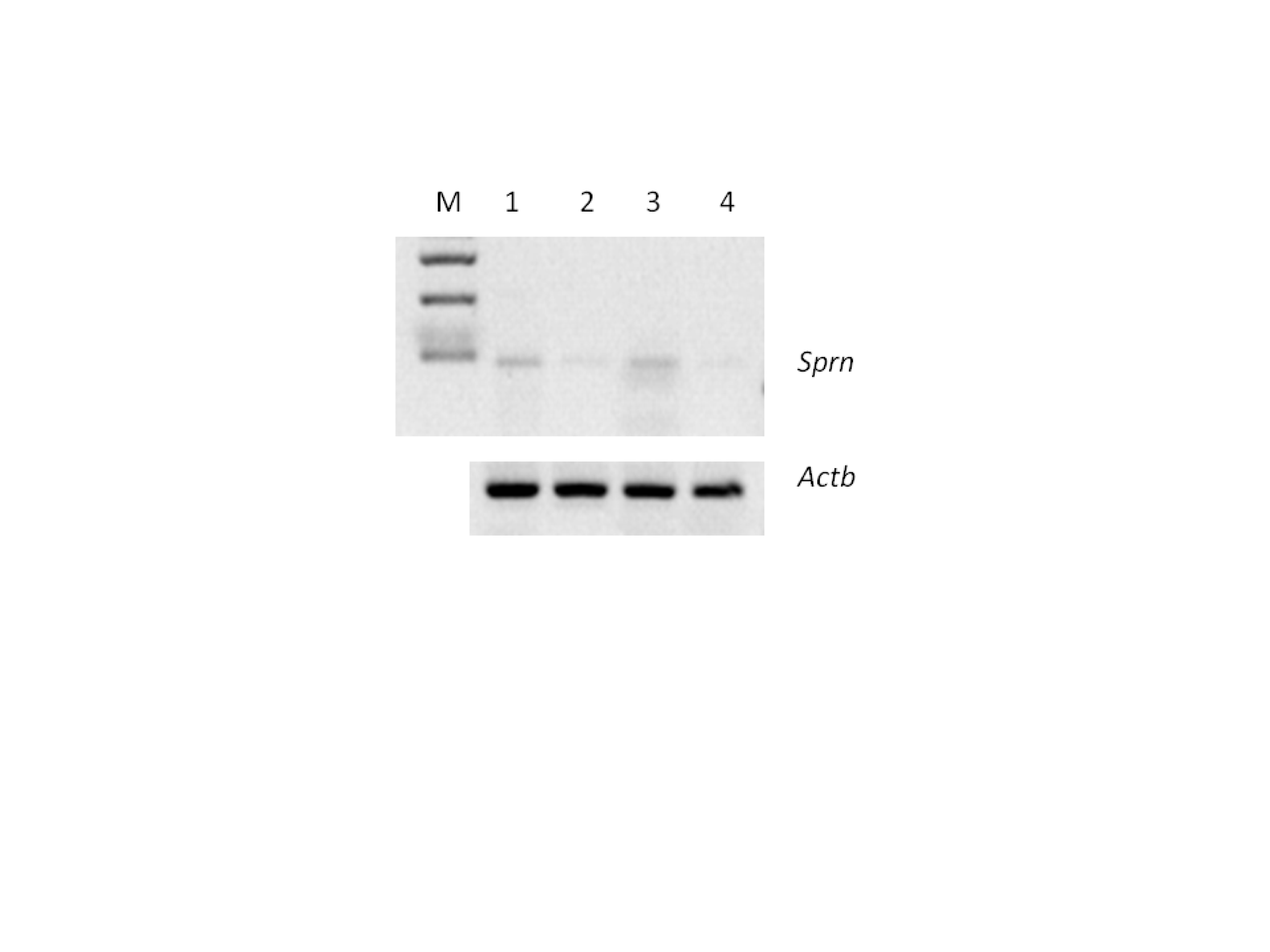

Supplement: Figure S2 — Evidence for Sprn downregulation. RT-PCR was performed on total RNA isolated from pooled E7.5 embryos. The used oligonucleotides and PCR conditions were as previously described (25). Actb: actin control RT-PCR. M: 1 kb ladder molecular weight marker (InVitrogen). 1: FVB/N embryos. 2: LS2-injected FVB/N embryos. 3: FVB/N PrnpKO embryos. 4: LS1-injected FVB/N Prnp KO embryos. (TIF) [file pone.0041959.s002.tif]

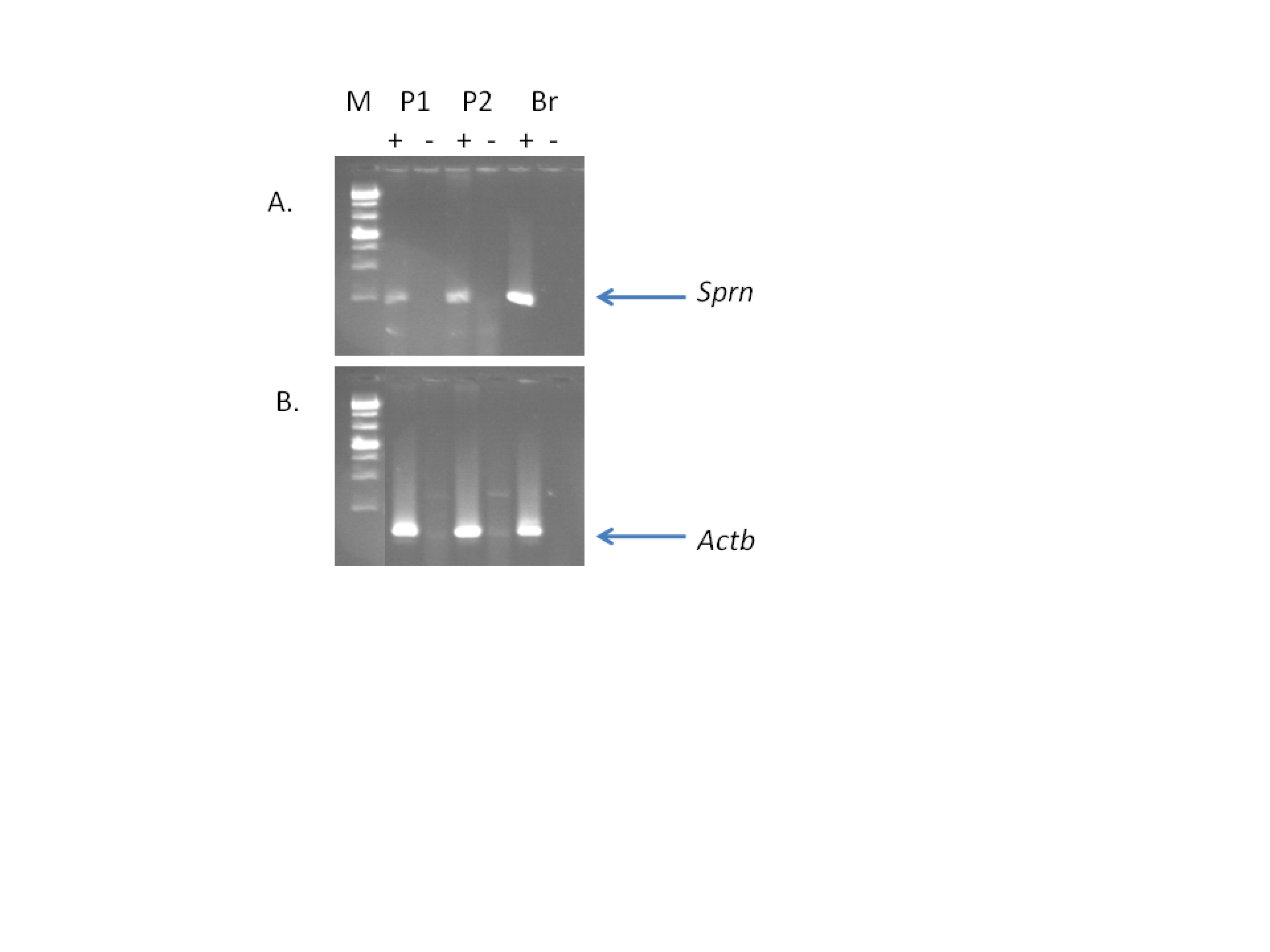

Supplement: Figure S3 — Evidence for Sprn expression in mouse placenta. RT-PCRs were performed on total RNA isolated from E12 mouse placenta embryos (P1 and P2) and adult brain (Br). The used oligonucleotides and PCR conditions were as previously described (25). Actb: actin control RT-PCR. M: 1 kb ladder molecular weight marker (InVitrogen). + with reverse transcriptase. – without reverse transcriptase. (TIF) [file pone.0041959.s003.tif]
