# Supplementary material for: Prion Protein and Shadoo Are Involved in Overlapping Embryonic Pathways and Trophoblastic Development
Source: PLoS One. 2012 Jul 30;7(7):e41959. doi: 10.1371/journal.pone.0041959 (PMC3408428; doi:10.1371/journal.pone.0041959)
Supplement: Table S1 — Complete list of genes differentially expressed in mouse embryos of various genotypes at E6.5 and E7.5. The genotypes of the compared embryos are indicated in the top line. Upregulated genes are in green. Downregulated genes are in red. Indicated numbers in each case refer to the observed fold ratio (p Value). For data involving Sprn knockdown experiments, in each case the first set of numbers is that observed with LS1, the second set is that observed with LS2. ??: upregulation of the gene with no observed expression in the control genotype. (DOC) [file pone.0041959.s004.doc]

| Name | Gene_ID | E7,5 *Prnp* KO versus E7,5 WT | E7,5 *Sprn* KD versus E7,5 WT | E7,5 *Prnp* KO *Sprn* KD versus E7,5 *WT* | E7,5 *Prnp* KO *Sprn* KD versus E7,5 *Prnp* KO | E7,5 *Prnp* KO *Sprn* KD versus E7,5 *Sprn* KD | E6,5 *Prnp* KO *Sprn* KD versus E6,5 *Sprn* KD | E6,5 *Prnp* KO *Sprn* KD versus E6,5 *Prnp* KO | E6,5 *Prnp* KO *Sprn* KD versus E6,5 *Prnp* WT | E6,5 *Sprn* KD versus E6,5 WT | E6,5 *Prnp* KO versus E6,5 WT | Name | Gene_ID | E7,5 *Prnp* KO versus E7,5 WT | E7,5 *Sprn* KD versus E7,5 WT | E7,5 *Prnp* KO *Sprn* KD versus E7,5 *WT* | E7,5 *Prep* KO *Sprn* KD versus E7,5 *Prnp* KO | E7,5 *Prnp* KO *Sprn* KD versus E7,5 *Sprn* KD | E6,5 *Prnp* KO *Sprn* KD versus E6,5 *Sprn* KD | E6,5 *Prnp* KO *Sprn* KD versus E6,5 *Prnp* KO | E6,5 *Prnp* KO *Sprn* KD versus E6,5 *Prnp* WT | E6,5 *Sprn* KD versus E6,5 WT | E6,5 *Prnp* KO versus E6,5 WT |
| --- | --- | --- | --- | --- | --- | --- | --- | --- | --- | --- | --- | --- | --- | --- | --- | --- | --- | --- | --- | --- | --- | --- | --- |
| A2m | gi|31342256|ref|NM_175628.2| | 0.1(3.6E-35) | 0.12(4E-05)  0.07(0.002) | 0.1(0.0006)  0.06(2E-10) |  |  |  |  |  |  |  | Add3 | gi|31542110|ref|NM_013758.2| | 0.39(0.02) |  | 0.2(0.02)  0.18(0.001) |  |  |  |  |  |  |  |
| Lbp | gi|113865990|ref|NM_008489.2| | 0.09(5.7E-35) |  | 0.08(3.4E-08)  0.03(8E-28) |  |  |  |  |  |  |  | Tnfaip3 | gi|31543879|ref|NM_009397.2| | 0.03(0.02) |  |  |  |  |  |  |  |  |  |
| Havcr2 | gi|21703359|ref|NM_134250.1| | 0.05(  1.6E-33) | 0.1(2.4E-05)  0.06(0.0004) | 0.08(1.4E-05)  0.05(1.8E-09) |  |  |  |  |  |  |  | Klk14 | gi|33469048|ref|NM_174866.1| | 0.07(0.02) |  | 0.02(0.04)  0.04(0.01) |  |  |  |  |  |  |  |
| Ly6c2 | gi|149266692|ref|XM_001477071.1| | 0.09(1.6E-30) | 0.15(0.0006)  0.08(0.002) | 0.15(0.003)  0.04(3.3E-11) |  |  |  |  |  |  |  | Sned1 | gi|40254324|ref|NM_172463.3| | 0.3(0.02) |  | 0.16(0.03)  0.09(5E-05) |  |  |  |  |  |  |  |
| misc | gi|94377878|ref|XR_005046.1| | 0.09(  1.3E-29) | 0.1(2.5E-06)  0.06(0.002) | 0.09(0.0001)  0.06(3.6E-12) |  |  |  |  |  |  |  |  | gi|149233590|ref|XM_001472850.1| | 0.4(0.02) |  |  |  |  |  |  |  |  |  |
| Slc6a12 | gi|141802667|ref|NM_133661.2| | 0.06(  3.2E-27) | 0.08(2.5E-09)  0.06(0.0001) | 0.08(3.2E-07)  0.06(3E-13) |  |  |  |  |  |  |  | Gpnmb | gi|142371734|ref|NM_053110.3| | 0.3(0.02) |  |  |  |  |  |  |  |  |  |
| Ly6c2 | gi|149266694|ref|XM_001477088.1| | 0.09(1.8E-26) | 0.15(0.0006)  0.06(0.0005) | 0.11(0.0001)  0.05(9.6E-11) |  |  |  |  |  |  |  | Cdx4 | gi|141802151|ref|NM_007674.2| | 0.25(0.02) |  | 0.08(0.004)  0.06(9.8E-06) |  |  |  |  |  |  |  |
| Ly6Cc1 | gi|58331141|ref|NM_010741.2| | 0.08(3.9E-26) | 0.1(1.5E-07)  0.06(0.0002) | 0.1(1.7E-05)  0.05(6.6E-14) |  |  |  |  |  |  |  | Acvr1c | gi|141802377|ref|NM_001033369.2| | 3.4(0.02) |  |  |  |  |  |  |  |  | 0 .2(0.04) |
| Hsd11b1 | gi|113680660|ref|NM_001044751.1| | 0.05(1E-25) |  | 0.07(2.5E-05)  0.06(3.5E-08) |  |  |  |  |  |  |  | Ctsk | gi|142352209|ref|NM_007802.3| | 0.39(0.02) |  |  |  |  |  | 4.8(0.02)  5.6(0.001) | 14.2(1.8E-07)  16.9(1.9E-34) | 25.5(8.3E-25)  16.7(1.4E-29) |  |
| Slpi | gi|141801540|ref|NM_011414.2| | 0.03(1.2E-24) | 0.12(0.0007)  0.05(0.0004) | 0.05(3.2E-07)  0.03(2.6E-10) |  |  |  |  |  |  |  | Pdgfrl | gi|42476141|ref|NM_026840.2| | 0.19(0.02) |  |  |  |  |  |  |  |  |  |
| Ly6A | gi|31981636|ref|NM_010738.2| | 0.09(5.4E-23) | 0.2(0.02)  0.09(0.01) | 0.12(0.002)  0.05(7E-22) |  |  |  |  |  |  |  | Timp3 | gi|119637812|ref|NM_011595.2| | 0.4(0.03) |  |  |  |  |  |  |  | 4.5(8.2E-05)  3.4(0.0004) |  |
| Mfap5 | gi|118131047|ref|NM_015776.2| | 0.13(5.8E-23) | 0.22(0.02)  0.09(0.006) | 0.17(0.01)  0.06(4E-16) |  |  |  |  |  |  |  | Mmp7 | gi|144227218|ref|NM_010810.3| | 7.8(0.03) | 545(3E-35)  65(2.7E-11) |  |  | 94.8(6E-59)  0.003(1.9E-16) |  |  | 19.6(3.6E-09)  8.2(7.6E-15) |  | 6.4(0.003) |
| Emcn | gi|8393724|ref|NM_016885.1| | 0.06(1E-22) | 0.16(0.001)  0.06(0.0003) | 0.14(0.001)  0.04(5.8E-18) |  |  |  |  |  |  |  |  | gi|149261822|ref|XM_001472216.1| | 0.1(0.03) |  |  |  |  |  |  |  |  |  |
| Sfrp5 | gi|31560420|ref|NM_018780.2| | 0.11(1E-22) | 0.23(0.02)  0.07(0.0004) | 0.18(0.007)  0.13(1.3E-05) |  |  |  |  |  |  |  | Trim16 | gi|70778827|ref|NM_053169.2| | 0.2(0.03) |  |  |  |  |  |  |  |  |  |
| Tdo2 | gi|31982696|ref|NM_019911.2| | 0.09(8.6E-22) | 0.1(1.52E-05)  0.1(0.02) | 0.14(0.01)  0.06(7.4E-11) |  |  |  |  |  |  |  | SecTm1b | gi|114145547|ref|NM_026907.3| | 0(0.03) |  |  |  |  |  |  |  |  |  |
| Psca | gi|21312315|ref|NM_028216.1| | 0.07(8.6E-22) | 0.15(0.0002)  0.06(0.0002) | 0.11(7.1E-05)  0.08(3.1E-08) |  |  |  |  |  |  |  | Rpph1 | gi|46402165|ref|NR_002142.1| | 3(0.03) |  |  |  |  |  | 9(1.4E-05)  5.6(0.005) |  | 0.28(0.03)  0.34(0.02) | 0.1(0.0006) |
| Dcn | gi|142383942|ref|NM_007833.3| | 0.11(4.3E-20) | 0.18(0.001)  0.07(0.0003) | 0.11(7E-05)  0.03(1.4E-15) |  |  |  |  |  |  |  | Ednrb | gi|141802494|ref|NM_007904.3| | 0.37(0.03) |  |  |  |  |  |  | 6.7(0.004)  4.6(1.1E-05) |  |  |
| Dmkn | gi|142375786|ref|NM_172899.3| | 0.1(1E-19) | 0.13(6.1E-06)  0.06(8E-05) | 0.12(7E-05)  0.06(2.1E-11) |  |  |  |  |  |  |  | Tm4sf1 | gi|88900519|ref|NM_008536.3| | 0.18(0.03) |  |  |  |  |  |  |  |  |  |
| Tnfrsf11b | gi|113930715|ref|NM_008764.3| | 0.09(1.7E-18) | 0.04(4.5E-13)  0.02(4E-09) | 0.04(1.7E-20)  0.03(2.5E-14) |  |  |  |  |  |  |  | Irak3 | gi|142380077|ref|NM_028679.3| | 0.26(0.03) |  |  |  |  |  |  |  |  |  |
| Des | gi|33563249|ref|NM_010043.1| | 0.09(9,5E-18) |  | 0.15(0.04)  0.06(5.7E-11) |  |  |  |  |  |  |  |  | gi|142360631|ref|NM_178737.4| | 2.8(0.03) |  |  | 0.12(3.3E-07)  0.08(2E-18) |  |  |  |  |  |  |
| Dmkn | gi|50284531|ref|NM_028618.1| | 0.1(1.2E-17) | 0.14(2.9E-05)  0.05(3.3E-05 | 0.12(0.0001)  0.06(1.5E-12) |  |  |  |  |  |  |  | Penk | gi|141801808|ref|NM_001002927.2| | 3.9(0.03) |  |  |  |  |  |  | 6.5(0.005)  3.1(0.01) |  |  |
| Lyve1 | gi|118131124|ref|NM_053247.4| | 0.06(5.2E-17) | 0.06(7.7E-09)  0.02(1.9E-06) | 0.06(1.3E-06)  0.02(8.9E-14) |  |  |  |  |  |  |  |  | gi|149264283|ref|XM_001477652.1| | 0.25(0.03) |  |  |  |  |  |  |  |  |  |
| Ly-6c | gi|149266926|ref|XM_001475001.1| | 0.09(6.2E-17) | 0.18(0.01)  0.09(0.007) | 0.12(0.02)  0.07(1.5E-07) |  |  |  |  |  |  |  | Bdnf | gi|114326458|ref|NM_001048142.1| | 0,16(0.03) |  |  |  |  |  |  |  |  |  |
| Ptrf | gi|71043973|ref|NM_008986.2| | 0.12(6.8E-17) | 0.13(1.3E-05)  0.006(0.0002) | 0.06(2.5E-10)  0.04(3E-14) |  |  |  |  |  |  | 13.7(8E-15) | Ldhd | gi|34328378|ref|NM_027570.3| | 0.11(0.03) |  |  |  |  |  |  |  |  |  |
| Dio3 | gi|111494233|ref|NM_172119.2| | 0.13(1E-16) | 0.16(0.0003)  0.13(0.01) | 0.19(0.01)  0.06(3.6E-12) |  |  |  |  |  |  |  | Ahnak1 | gi|61743960|ref|NM_009643.1| | 0.42(0.03) |  |  |  |  |  |  |  |  |  |
| Crip1 | gi|123701561|ref|NM_007763.3| | 0.14(1,9E-16) | 0.2(0.004)  0.1(0.004) | 0.19(0.01)  0.09(1.5E-08) |  |  |  |  |  |  |  | CD55 | gi|114326521|ref|NM_010016.2| | 0.34(0.03) |  |  |  |  |  |  |  |  |  |
| Gatm | gi|142354911|ref|NM_025961.3| | 0.1(7.9E-16) |  |  |  |  |  |  |  |  |  | Degs2 | gi|142345826|ref|NM_027299.3| | 6,75(0.03) |  |  |  |  |  |  | 34(2.4E-10)  21.4(4.6E-13) | 34.8(3.2E-25)  20.3(4E-17) |  |
| Hspb7 | gi|142351368|ref|NM_013868.3| | 0.01(3E-15) | 0.03(6.2E-07)  0.08(0.03) | 0.02(3.3E-08)  0.02(2.9E-09) |  |  |  |  |  | 0.08(0.01)  0.04(0.0003) |  |  | gi|149256063|ref|XM_0389.101479| | 0.3(0.03) |  |  |  |  |  |  |  |  |  |
| Tgfbr2 | gi|27363473|ref|NM_009371.2| | 0.16(4.8E-14) |  | 0.2(0.03)  0.06(9.8E-12) |  |  |  |  |  |  |  | Mixl1 | gi|31543252|ref|NM_013729.2| | 2.8(0.03) |  |  |  |  |  | 0.05(1.5E-13)  0.2(0.004) |  |  |  |
| Prkg2 | gi|142372298|ref|NM_008926.3| | 0.12(1.3E-13) |  | 0.11(6.4E-05)  0.09(5.4E-08) |  |  |  |  |  |  |  | Slco1a6 | gi|12963796|ref|NM_023718.1| | 11.4(0.03) |  |  |  |  |  |  |  |  |  |
| Hsd11b1 | gi|113680740|ref|NM_008288.2| | 0.05(2.4E-13) |  | 0.06(2E-05)  0.06(5.4E-08) |  |  |  |  |  |  |  | Corin | gi|142374600|ref|NM_016869.2| | 0.05(0.03) |  |  |  |  |  |  |  |  |  |
| Pdzk1ip1 | gi|141802816|ref|NM_026018.2| | 0.09(1.2E-12) | 0.22(0.03)  0.11(0.01) | 0.12(0.001)  0.1(1.9E-06) |  |  |  |  |  |  |  |  | gi|149251615|ref|XM_001471696.1| | 0.18(0.03) |  |  |  |  |  |  |  |  |  |
| Anxa8 | gi|142377277|ref|NM_013473.3| | 0.07(4E-12) | 0.14(0.005)  0.1(0.0001) | 0.09(0.001)  0.09(4.9E-05) |  |  |  |  |  |  |  |  | gi|149256635|ref|XM_001480672.1| | 0.33(0.03) |  |  |  |  |  |  |  |  |  |
| Ptges | gi|46852157|ref|NM_022415.2| | 0.11(1.7E-11) |  | 0.16(0.008)  0.12(9.8E-06) |  |  |  |  |  |  |  |  | gi|149263982|ref|XM_904319.2| | 0.37(0.03) |  |  |  |  |  |  |  |  |  |
| Gda | gi|87299644|ref|NM_010266.2| | 0.14(2.5E-11) |  |  |  |  |  |  |  |  |  | Cubn | gi|124487347|ref|NM_001081084.1| | 2.3(0.03) |  |  |  |  |  |  |  |  |  |
| Acss1 | gi|18034772|ref|NM_080575.1| | 0.1(5.3E-11) |  |  |  |  |  |  |  |  |  | Usp53 | gi|142371445|ref|NM_133857.2| | 0.38(0.03) |  |  |  |  |  |  |  |  |  |
| Fbn1 | gi|118197276|ref|NM_007993.2| | 0.14(6.6E-11) |  | 0.08(9.3E-07)  0.04(5E-16) |  |  |  |  |  |  |  | Zbtb20 | gi|9790132|ref|NM_019778.1| | 0.26(0.03) |  |  |  |  |  |  |  |  |  |
| Tmem176b | gi|142372371|ref|NM_023056.3| | 0.15(8.3E-11) |  | 0.17(0.01)  0 .09(6.8E-08) |  |  |  |  |  |  |  | Pou5f1 | gi|125490391|ref|NM_013633.2| | 2.5(0.03) |  |  |  |  |  |  |  |  |  |
| Smoc2 | gi|11612492|ref|NM_022315.1| | 0.14(1.1E-10) | 0.12(5.2E-05)  0.1(0.005) | 0.09(2E-05)  0.04(2.2E-11) |  |  |  |  |  |  |  |  | gi|149269617|ref|XM_001473270.1| | 0.33(0.03) |  |  |  |  |  |  |  |  |  |
| Sorbs2 | gi|124301205|ref|NM_172752.3| | 0.18(1.1E-10) |  | 0.17(0.006)  0.12(4.3E-06) |  |  |  |  |  |  |  | Serpina1e | gi|76881810|ref|NM_009247.2| | 32.6(0.03) | 898(1.6E-20)  136(0.02) |  |  | 23.7(6E-9)  0.04(0.01) |  | 821(9.8E-27)  106(2.6E-11) | 365(9E-19)  48.3(4.3E-09) |  |  |
| Ceacam10 | gi|142352593|ref|NM_007675.3| | 0.15(2.8E-10) | 0.2(0.009)  0.13(0.02) | 0.18(0.01)  0.12(1.9E-05) |  |  |  |  |  |  |  | Aqp1 | gi|6680709|ref|NM_007472.1| | 0.1(0.03) |  |  |  |  |  |  |  |  |  |
| Gpr115 | gi|149268852|ref|XM_894986.3| | 0.09(2.8E-10) |  | 0.09(0.0002)  0.08(5.6E-06) |  |  |  |  |  |  |  | Casp12 | gi|142367382|ref|NM_009808.3| | 0.17(0.03) |  |  |  |  |  |  |  |  |  |
|  | gi|31342510|ref|NM_178098.2| | 0.19(2.8E-10) |  |  |  |  |  |  |  |  |  |  | gi|142360223|ref|NM_024283.2| | 12.6(0.04) |  |  |  |  |  |  |  |  |  |
| Gpx3 | gi|6680076|ref|NM_008161.1| | 0.18(3.1E-10) |  |  |  |  |  |  |  |  |  | CD36 | gi|142363407|ref|NM_007643.3| | 0.24(0.04) |  |  |  |  |  |  |  |  |  |
| Mgp | gi|123701577|ref|NM_008597.3| | 0.2(3.6E-10) |  |  |  |  |  |  |  |  |  | Lgr4 | gi|124430558|ref|NM_172671.2| | 0.4(0.04) |  |  |  |  |  |  |  |  |  |
| Hoxa10 | gi|6680242|ref|NM_008263.1| | 0.2(6.2E-10) |  | 0.14(0.001)  0.1(8.9E-08) |  |  |  |  |  |  |  | Apob | gi|110625670|ref|NM_009693.1| | 2.3(0.04) |  |  |  |  |  |  |  |  |  |
| Olfml3 | gi|86439988|ref|NM_133859.2| | 0.17(1.6 E-09) |  | 0.13(0.001)  0.04(7.4E-23) |  |  |  |  |  |  |  | Alox5 | gi|116686109|ref|NM_009662.2| | 0.09(0.04) |  |  |  |  |  |  |  |  |  |
| Prap1 | gi|6678510|ref|NM_009475.1| | 11.3(1,7E-09) | 124(2E-33)  55(3E-10) |  |  | 36.8(9E-43)  0.005(6E-19) |  |  | 13.5(3.4E-07)  5.6(2.5E-09) |  |  | C2 | gi|7304936|ref|NM_013484.1| | 0.17(0.04) |  |  |  |  |  |  |  |  |  |
| Nipal1 | gi|124487088|ref|NM_001081205.1| | 0.17(2.3E-09) |  |  |  |  |  |  |  |  |  | Bche | gi|124487349|ref|NM_009738.3| | 0.15(0.04) |  |  |  |  |  |  |  |  |  |
| Gpr115 | gi|149269311|ref|XM_001476141.1| | 0.07(2.3E-09) |  | 0.06(0.0009)  0.07(4.9E-05) |  |  |  |  |  |  |  | Hba-x | gi|142350815|ref|NM_010405.3| | 0.18(0.04) |  | 0.01(0.0001)  0.02(3.3E-05) |  |  |  |  |  |  |  |
| SqrdI | gi|31981548|ref|NM_021507.4| | 0.13(2.6E-09) |  | 0.18(0.02)  0.09(1.3E-06) |  |  |  |  |  |  |  | Fads3 | gi|70887800|ref|NM_021890.3| | 0.29(0.04) |  |  |  |  |  |  |  |  |  |
| Col5a2 | gi|86613789|ref|NM_007737.2| | 0.18(2.7E-09) | 0.2(0.01)  0.14(0.03) | 0.16(0.006)  0.09(3.4E-07) |  |  |  |  |  |  |  | Tshz2 | gi|61097921|ref|NM_080455.1| | 0.27(0.04) |  |  |  |  |  |  |  |  |  |
| Ano1 | gi|110835695|ref|NM_178642.4| | 0.15(5.7E-09) |  | 0.14(0.003)  0.05(4.4E-11) |  |  |  |  |  |  |  |  | gi|149250374|ref|XM_001475472.1| | 0.38(0.04) |  |  |  |  |  |  |  |  |  |
| Rmrp | gi|32526884|ref|NR_001460.1| | 4.2(6.6E-09) |  |  |  |  |  | 7.3(0.0008)  5.7(0.0006) |  | 0.16(6.5E-09)  0.24(1.2E-06) | 0.06(5E-24) |  | gi|21311852|ref|NM_028747.1| | 0.2(0.04) |  |  |  |  |  |  |  |  |  |
| Mtap7d2 | gi|124487456|ref|NM_001081124.1| | 0.18(8.8E-09) |  |  |  |  |  |  |  |  |  | LOC677333 | gi|149269299|ref|XR_031734.1| | 2.45(0.04) |  |  |  |  |  |  |  |  | 0.2(0.01) |
| CD34 | gi|19526791|ref|NM_133654.1| | 0.15(1.1E-08) |  | 0.19(0.03)  0.09(1.8E-06) |  |  |  |  |  |  |  | Hoxd10 | gi|141802469|ref|NM_013554.3| | 0.28(0.04) |  |  |  |  |  |  | 7.6(0.009)  5(0.001) |  |  |
| Suox | gi|31343458|ref|NM_173733.2| | 0.15(1.2E-08) |  |  |  |  |  |  |  |  |  | Rgs2 | gi|141802972|ref|NM_009061.3| | 0.4(0.04) |  |  |  |  |  |  |  |  |  |
| Angpt2 | gi|118131014|ref|NM_007426.3| | 0.19(1.3E-08) | 0.25(0.05)  0.13(0.02) | 0.21(0.04)  0.14(4.2E-05) |  |  |  |  |  | 3.7(0.02)  3.4(0.004) |  | Hoxd8 | gi|112807183|ref|NM_008276.2| | 0.13(0.04) |  |  |  |  |  |  |  |  |  |
| Fbln2 | gi|124517692|ref|NM_001081437.1| | 0.14(1.3E-08) | 0.22(0.04)  0.14(0.04) | 0.12(0.002)  0.12(8.7E-05) |  |  |  |  |  |  |  | Eomes | gi|83921571|ref|NM_010136.2| | 2.3(0.04) |  | 4.4(0.03)  8.2(2.8E-07) |  |  |  |  |  |  |  |
| Fbln2 | gi|124517703|ref|NM_007992.2| | 0.14(1.3E-08) |  |  |  |  |  |  |  |  |  | Krt84 | gi|33563237|ref|NM_008474.1| | 0.16(0.04) |  |  |  |  |  |  |  |  |  |
| Tmem176a | gi|142353616|ref|NM_025326.3| | 0.15(1.3E-08) |  |  |  |  |  |  |  |  |  | Hist1h2ao | gi|149263930|ref|XM_978341.2| | 2.9(0.05) |  |  |  |  |  |  |  |  |  |
| Abat | gi|37202120|ref|NM_172961.2| | 0.14(1.3E-08) |  |  |  |  |  |  |  |  |  | Lum | gi|6678739|ref|NM_008524.1| | 4.6(0.05) |  |  |  |  | 6.1(0.01)  4.5(0.0004) |  |  |  |  |
|  | gi|149266696|ref|XM_001477102.1| | 0.1(3.5E-08) |  | 0.14(0.03)  0.04(1.8E-07) |  |  |  |  |  |  |  | Tacc1 | gi|110681726|ref|NM_177089.4| | 0.4(0.05) |  |  |  |  |  |  |  |  |  |
|  | gi|110626043|ref|NM_029142.1| | 0.11(4.6E-08) |  |  |  |  |  |  |  |  |  | Rufy3 | gi|31542481|ref|NM_027530.2| |  |  |  |  |  |  | 6(0.001)  4.7(0.006) |  |  | 0.07(  1E-11) |
| Sfrp4 | gi|141803390|ref|NM_016687.2| | 0.18(5.5E-08) |  |  |  |  |  |  | 8.2(0.0007)  4.1(0.0004) |  |  | Magel2 | gi|94380522|ref|XM_622091.3| |  |  |  |  |  |  |  | 0.15(0.02)  0.1(8.1E-12) |  | 0.03(  1.8E-11) |
| Cldn1 | gi|142350296|ref|NM_016674.3| | 0.06(8.2E-08) |  | 0.06(0.001)  0.04(2.8E-06) |  |  |  |  |  |  |  | Speer2 | gi|144925922|ref|NM_173069.2| |  |  |  |  |  |  |  |  |  | 0.01(  3.4E-9) |
| Mal | gi|141801061|ref|NM_010762.4| | 0.11(8.8E-08) |  | 0.13(0.02)  0.02(1.8E-06) |  |  |  |  |  |  |  | Myoz1 | gi|134032009|ref|NM_021508.3| |  |  |  |  |  |  |  |  |  | 0.05(  2.7E-7) |
| S100a4 | gi|33859623|ref|NM_011311.1| | 0.16(9.5E-08) | 0.21(0.03)  0.14(0.05) |  |  |  |  |  |  |  |  | Fam186b | gi|124486942|ref|NM_001081254.1| |  |  |  |  |  |  |  |  |  | 0.02(  3.2E-7) |
| Ptgs1 | gi|144227245|ref|NM_008969.3| | 0.1(1E-07) |  | 0.1(0.003)  0.1(0.0003) |  |  |  |  |  |  |  | LOC100043825 | gi|149265870|ref|XM_001481034.1| |  |  |  |  |  |  |  |  |  | 0.05(  6E-6) |
| Mt3 | gi|7305286|ref|NM_013603.1| | 0.13(1.6E-07) |  |  |  |  |  |  |  |  |  | LOC100046120 | gi|149266011|ref|XM_001475611.1| |  |  |  |  |  |  |  | 20.4(1.3E-11)  7.3(7.8E-09) |  | 9.8(  0.0001) |
| Kcnd3 | gi|86991433|ref|NM_001039347.1| | 0.03(1.6E-07) |  |  |  |  |  |  |  |  |  | Prl2a1 | gi|9910513|ref|NM_019991.1| |  |  |  |  |  |  |  | 0.04(1.2E-23)  0.16(4.3E-12) | 0.18(7.6E-08)  0.27(8E-07) | 0.12(  0.0001) |
| Srd5a1 | gi|87044894|ref|NM_175283.3| | 0.2(2.4E-07) |  | 0.16(0.006)  0.15(0.0002) |  |  |  |  |  |  |  | Scrt2 | gi|149250060|ref|XM_619828.4| |  |  |  |  |  |  |  |  |  | 0.06(  0.0003) |
| Ctso | gi|118130454|ref|NM_177662.2| | 0.18(2.5E-07) | 0.12(0.0002)  0.09(0.004) | 0.09(7E-05)  0.06(1.2E-09) |  |  |  |  |  |  |  | Pcdh19 | gi|149272244|ref|XM_205287.6| |  |  |  |  |  |  |  |  |  | 0.06(  0.0007) |
| Htra3 | gi|110815868|ref|NM_0320127.| | 0.12(3E-07) |  |  |  |  |  |  |  |  |  | LOC633979 | gi|149260699|ref|XR_032514.1| |  |  |  |  |  |  |  |  |  | 0.03(  0.0008) |
| Gpihbp1 | gi|58037120|ref|NM_026730.1| | 0.13(3.6E-07) |  |  |  |  |  |  |  |  |  | Indol1 | gi|22122378|ref|NM_145949.1| |  |  |  |  |  |  |  |  |  | 0.06(  0.0008) |
| Slc2a12 | gi|142387928|ref|NM_178934.3| | 0.19(3.9E-07) | 0.18(0.007)  0.15(0.05) | 0.12(0.0009)  0.07(6.4E-08) |  |  |  |  |  |  |  | Pcdh19 | gi|94408163|ref|XM_975492.1| |  |  |  |  |  |  |  |  |  | 0.06(  0.001) |
| Ly6i | gi|21356650|ref|NM_020498.1| | 0.13(5E-07) |  | 0.11(0.003)  0.07(2.2E-06) |  |  |  |  |  |  |  | Cdh22 | gi|31341388|ref|NM_174988.2| |  |  |  |  |  |  |  |  | 0.07(0.001)  0.1(0.0002) | 0.04(  0.001) |
| Prps2 | gi|141802916|ref|NM_026662.3| | 0.27(8.5E-07) |  |  |  |  |  |  |  |  |  | Pax8 | gi|118130210|ref|NM_011040.3| |  |  |  |  |  |  |  | 27(0.0001)  7.6(0.03) |  | 16.5(  0.001) |
| Ptn | gi|118130571|ref|NM_008973.2| | 0.26(8.9E-07) | 0.21(0.006)  0.12(0.02) | 0.18(0.01)  0.04(1.9E-33) |  |  |  |  |  |  |  | EG666182 | gi|94363835|ref|XM_991070.1| |  |  |  |  |  |  |  |  |  | 0.08(  0.003) |
| Sat1 | gi|42476316|ref|NM_009121.3| | 0.27(1E-06) |  |  |  |  |  |  |  |  |  | Hist2h3c2-ps | gi|34328342|ref|NM_054045.2| |  |  |  |  |  |  |  |  |  | 0.13(  0.003) |
| Il6ra | gi|110431355|ref|NM_010559.2| | 0.17(1.4E-06) |  |  |  |  |  |  |  |  |  | Hist2h2bb | gi|68226432|ref|NM_175666.2| |  |  |  |  |  |  |  |  |  | 0.1(  0.004) |
| Pglyrp1 | gi|118130401|ref|NM_009402.2| | 0.09(1.4E-06) |  |  |  |  |  |  |  |  |  | Hist2h3c1 | gi|30061400|ref|NM_178216.1| |  |  |  |  |  |  |  |  |  | 0.13(  0.004) |
| Ampd3 | gi|66792801|ref|NM_009667.2| | 0.21(1.4E-06) |  |  |  |  |  |  |  |  |  | Ddx58 | gi|45598376|ref|NM_172689.2| |  |  |  |  |  |  |  |  |  | 0.16(  0.004) |
|  | gi|142376574|ref|NM_177599.3| | 0.01(5.4E-09) |  | 0.02(0.005)  O.06(O.003) |  |  |  |  |  |  |  | Hmx2 | gi|110625930|ref|NM_145998.2| |  |  |  |  |  |  |  |  |  | 0.09(  0.005) |
| Serping1 | gi|142370111|ref|NM_009776.2| | 0.26(6.8E-09) |  |  |  |  |  |  |  |  |  | LOC100046008 | gi|149250380|ref|XM_001475350.1| |  |  |  |  |  |  |  |  | 0.13(0.01)  0.15(0.002) | 0.07(  0.005) |
| Dpep1 | gi|6681216|ref|NM_007876.1| | 0.12(1.3E-08) |  |  |  |  |  |  |  |  |  | LOC627701 | gi|94372774|ref|XM_892400.2| |  |  |  |  |  |  |  |  |  | 0.17(  0.005) |
| Sgk1 | gi|6755489|ref|NM_011361.1| | 0.28(1.8E-08) |  |  |  |  |  |  |  |  |  | AA474408 | gi|149258180|ref|XR_035227.1| |  |  |  |  |  |  |  |  |  | 0.17(  0.006) |
|  | gi|94399046|ref|XM_909906.2| | 0.12(1.9E-08) |  | 0.14(0.04)  0.04(5.9E-08) |  |  |  |  |  |  |  | Ado | gi|53749203|ref|NM_001005419.1| |  |  |  |  |  |  | 0.19(0.01)  0.18(0.001) |  |  | 5.9(0.006) |
| Srgn | gi|118129990|ref|NM_011157.2| | 0.29(2E-08) |  |  |  |  |  |  |  |  |  | Stac3 | gi|141803518|ref|NM_177707.3| |  |  |  |  |  |  |  |  |  | 0.07(0.006) |
| Epdr1 | gi|142372189|ref|NM_134065.3| | 0.21(2E-08) |  |  |  |  |  |  |  |  |  | Pcdh19 | gi|149272245|ref|XM_001473606.1| |  |  |  |  |  |  |  |  |  | 0.08(0.007) |
| Fstl1 | gi|141802756|ref|NM_008047.3| | 0.29(2.5E-08) |  |  |  |  |  |  |  |  |  | Dnajb5 | gi|40254371|ref|NM_019874.3| |  |  |  |  |  |  |  |  |  | 0.06(0.007) |
|  | gi|94400798|ref|XM_918758.2| | 0.1(2.5E-08) |  |  |  |  |  |  |  |  |  | Zfp109 | gi|9931981|ref|NM_020262.1| |  |  |  |  |  |  |  |  |  | 0.12(0.007) |
| Ctla2b | gi|149264174|ref|XM_001477566.1| | 0.29(3.5E-08) |  |  |  |  |  |  |  |  |  | Prlpa | gi|142364521|ref|NM_011165.2| |  |  |  |  |  |  |  | 0.05(3.2E-09)  0.13(3.5E-15) | 0.2(9.7E-06)  0.2(1.2E-08) | 0.18(0.007) |
| Ctla2a | gi|6681076|ref|NM_007796.1| | 0.24(4.3E-08) |  |  |  |  |  |  |  |  |  | Clu | gi|7304966|ref|NM_013492.1| |  |  |  |  |  |  |  | 12.5(2.1E-06)  4.5(0.0001) |  | 6(0.01) |
|  | gi|94400799|ref|XM_904281.2| | 0.12(4.7E-08) |  |  |  |  |  |  |  |  |  | LOC100046045 | gi|149270001|ref|XR_032266.1| |  |  |  |  |  |  |  |  |  | 0.04(0.01) |
| Tlr3 | gi|144227205|ref|NM_126166.3| | 0.16(5E-08) |  | 0.15(0.02)  0.07(6.4E-06) |  |  |  |  |  |  |  | AA474408 | gi|149257938|ref|XR_035336.1| |  |  |  |  |  |  |  |  |  | 0.2(0.01) |
| Cryaa | gi|113931666|ref|NM_013501.2| | 0.12(5.9E-08) |  | 0.14(0.04)  0.06(9.8E-06) |  |  |  |  |  |  |  | LOC677333 | gi|149268680|ref|XR_034279.1| |  |  |  |  |  |  |  |  |  | 0.18(0.01) |
|  | gi|110625808|ref|NM_027627.1| | 0.13(6.3E-08) | 0.17(0.04)  0.08(0.02) |  |  |  |  |  |  |  |  | LOC435145 | gi|94398806|ref|XM_487043.4| |  |  |  |  |  |  |  |  |  | 0.09(0.01) |
| Igfbp7 | gi|62990156|ref|NM_008048.2| | 0.25(3.3E-05) |  | 0.2(0.04)  0.08(3.5E-09) |  |  |  |  |  |  |  | Pfas | gi|149262172|ref|XM_908501.3| |  |  |  |  |  |  | 0.19(0.01)  0.24(0.03) |  |  | 5.4(0.01) |
| Parm1 | gi|118130163|ref|NM_145562.2| | 0.26(3.4E-05) |  |  |  |  |  |  |  |  |  | Hist2h3b | gi|30061346|ref|NM_178215.1| |  |  |  |  |  |  |  |  |  | 0.15(0.01) |
| Cldn10 | gi|144226219|ref|NM_021386.3| | 0.18(4.1E-05) |  | 0.17(0.04)  O.12(0.0004) |  |  |  |  |  |  |  | C330006A16 | gi|94366305|ref|XM_358556.5| |  |  |  |  |  |  |  |  |  | 5.5(0.01) |
| Cryab | gi|133892993|ref|NM_009964.2| | 0.3(4.9E-05) |  |  |  |  |  |  |  |  |  | C330006A16 | gi|94366195|ref|XM_913936.2| |  |  |  |  |  |  |  |  |  | 5.5(0.01) |
| Vldlr | gi|7305628|ref|NM_013703.1| | 0.2(4.9E-05) |  |  |  |  |  |  |  |  |  | LOC100048759 | gi|149274892|ref|XR_035041.1| |  |  |  |  |  |  |  | 7.5(0.007)  4.1(0.01) |  | 6.3(0.02) |
| Napsa | gi|6680551|ref|NM_008437.1| | 26.4(4.9E-05) | 124(1.2E-13)  63(0.0001) |  |  | 13.9(3.5E-10)  0.04(O.0002) |  |  |  |  | 9.15(  0.0002) | LOC676933 | gi|149251186|ref|XR_031924.1| |  |  |  |  |  |  |  |  |  | 28(0.02) |
| Cyp11b1 | gi|141803200|ref|NM_001033229.2| | 0.13(5.6E-05) | 0.07(0.0002)  0.1(0.05) | 0.02(3.2E-06)  0.03(1.8E-08) |  |  |  |  |  |  |  | Gm996 | gi|53749207|ref|NM_001005424.1| |  |  |  |  |  |  |  |  |  | 0.11(0.02) |
| Tmem154 | gi|31343187|ref|NM_177260.2| | 0.09(5.7E-05) |  |  |  |  |  |  |  |  |  | Tmem102 | gi|142360242|ref|NM_001033433.2| |  |  |  |  |  |  | 0.17(0.005)  0.16(0.001) |  |  | 6.1(0.02) |
|  | gi|94374638|ref|XM_485586.4| | 0.25(5.7E-05) |  |  |  |  |  |  |  |  |  | Pfas | gi|149261973|ref|XM_111232.8| |  |  |  |  |  |  |  |  |  | 5(0.02) |
| Nampt | gi|10946947|ref|NM_021524.1| | 0.3(5.3E-05) |  |  |  |  |  |  |  |  |  | Dscam | gi|118130729|ref|NM_031174.3| |  |  |  |  |  |  |  |  |  | 0.13(0.03) |
|  | gi|94400800|ref|XM_918770.2| | 0.11(9E-05) |  |  |  |  |  |  |  |  |  | Chsy3 | gi|124486748|ref|NM_001081328.1| |  |  |  |  |  |  |  |  |  | 0.08(0.03) |
| Ehd3 | gi|141802831|ref|NM_020578.2| | 0.23(0.0001) |  |  |  |  |  |  |  |  |  | AA474408 | gi|149257659|ref|XR_035373.1| |  |  |  |  |  |  |  |  |  | 0.2(0.03) |
|  | gi|149269704|ref|XM_001476237.1| | 0.22(0.0001) |  | 0.12(0.02)  0.19(0.008) |  |  |  |  |  |  |  | Mesp1 | gi|33469090|ref|NM_008588.1| |  |  |  |  |  |  |  | 0.02(1.3E-07)  0.1(9.7E-08) | 0.14(0.0001)  0.13(7.7E-08) | 0.17(0.03) |
| Adam12 | gi|117606340|ref|NM_007400.2| | 0.16(0.0001) |  |  |  |  |  |  |  |  |  | LOC633979 | gi|149260818|ref|XR_035504.1| |  |  |  |  |  |  |  |  |  | 0.08(0.03) |
|  | gi|94374423|ref|XM_908115.2| | 0.26(0.0001) |  |  |  |  |  |  |  |  |  | 5330417C22 | gi|85701809|ref|NM_001033304.1| |  | 53(9E-12)  19(0.02) |  |  | 39.5(4.7E-18)  0.1(0.04) |  |  | 11.7(6.5E-05)  4.3(0.006) |  | 6(0.03) |
| 4933425M15 | gi|31342302|ref|NM_175674.2| | 6(0.0002) |  |  |  |  |  |  |  | 0.19(0.005)  0.27(0.005) | 0.06(8.1E-06) | 170003OG11 | gi|94367656|ref|XM_130735.7| |  |  |  |  |  |  |  | 0.06(0.04)  0.06(0.003) |  | 0.08(0.03) |
| Fmo1 | gi|31981773|ref|NM_010231.2| | 0.31(0.0002) |  |  |  |  |  |  |  |  |  | Kcnc1 | gi|76677931|ref|NM_008421.2| |  |  |  |  |  |  |  |  |  | 0.1(0.03) |
|  | gi|149264700|ref|XR_032778.1| | 0.03(0.0002) |  |  |  |  |  |  |  |  |  | Rax | gi|89274178|ref|NM_013833.2| |  |  |  |  |  |  |  |  |  | 0.02(0.03) |
| Samhd1 | gi|46909601|ref|NM_018851.2| | 0.32(0.0003) |  |  |  |  |  |  |  |  |  | Scn3b | gi|144922642|ref|NM_178227.4| |  |  |  |  |  |  |  |  |  | 0.07(0.03) |
| Cxcl14 | gi|119392097|ref|NM_019568.2| | 0.24(0.0003) |  |  |  |  |  |  | 5.1(0.04)  4(0.0004) |  |  | LOC435145 | gi|94399138|ref|XM_987106.1| |  |  |  |  |  |  |  |  |  | 0.11(0.03) |
| Ugt1a7c | gi|141803423|ref|NM_201642.3| | 0.1(0.0004) |  |  |  |  |  |  |  |  |  | BC015286 | gi|142371927|ref|NM_198171.2| |  |  |  |  |  |  |  |  |  | 6.9(0.03) |
| Abp1 | gi|22095012|ref|NM_029638.1| | 0.32(0.0004) |  |  |  |  |  |  |  |  |  | LOC677333 | gi|149269258|ref|XR_031461.1| |  |  |  |  |  |  |  |  |  | 0.2(0.04) |
| Dtna | gi|95113657|ref|NM_207650.3| | 0.24(0.0004) |  |  |  |  |  |  |  |  |  | Hist4h4 | gi|28316745|ref|NM_175652.1| |  |  |  |  |  |  |  |  |  | 0.08(0.04) |
| Msln | gi|9256567|ref|NM_018857.1| | 0.01(0.0004) |  |  |  |  |  |  |  |  |  | Hist1h4h | gi|141802886|ref|NM_153173.2| |  |  |  |  |  |  |  |  |  | 0.12(0.04) |
| Tdgf1 | gi|134053944|ref|NM_011562.2| | 3.8(0.0004) | 7.6(2.5E-05)  6.5(0.03) | 7.6(0.0001)  21.3(1.3E-19) |  |  |  |  |  |  |  | Hist2h4 | gi|21361208|ref|NM_033596.1| |  |  |  |  |  |  |  |  |  | 0.11(0.04) |
|  | gi|149265290|ref|XM_910675.3| | 0.08(0.0005) |  | 0.08(0.04)  0.13(O.03) |  |  |  |  |  |  |  | LOC666964 | gi|94395360|ref|XM_987269.1| |  |  |  |  |  |  |  |  |  | 0.11(0.04) |
|  | gi|149256881|ref|XM_001478066.1| | 0.08(0.0006) |  |  |  |  |  |  |  |  |  | C3 | gi|126518316|ref|NM_009778.2| |  |  |  |  | 0.05(3.6E-27)  0.13(0.03) |  |  | 6.8(0.007)  3.9(0.003) |  | 5.2(0.04) |
| Ppp1r3c | gi|33468956|ref|NM_016854.1| | 0.05(0.0006) |  |  |  |  |  |  |  |  |  | Fam154b | gi|134288849|ref|NM_177894.4| |  |  |  |  |  |  |  |  |  | 0.07(0.04) |
| Prss28 | gi|115299757|ref|NM_053259.2| | 16.9(0.0006) | 228(2.2E-22)  352(1.7E-15) |  |  | 0.1(1.6E-24)  0.01(9E-17) |  | 19(3E-06)  13(4.4E-11) | 100.5(3.2E-31)  70(1.1E-73) | 74(6E-64)  3(0.03) | 5.2(0.04) | Mgat4c | gi|142385876|ref|NM_026243.3| |  |  |  |  |  |  |  |  |  | 15.5(0.04) |
| Atoh8 | gi|118130066|ref|NM_153778.3| | 0.21(0.0006) |  |  |  |  |  |  |  |  |  | LOC383326 | gi|149269761|ref|XR_033450.1| |  |  |  |  |  |  |  |  |  | 5.5(0.04) |
|  | gi|126506315|ref|NM_001081963.1| | 0.32(0.0006) |  |  |  |  |  |  |  |  |  | LOC636319 | gi|149270024|ref|XR_032953.1| |  |  |  |  |  |  |  |  |  | 5.5(0.04) |
| Igf1 | gi|133892662|ref|NM_010512.3| | 0.2(0.0006) |  |  |  |  |  |  |  |  |  | H2-M10.1 | gi|34328212|ref|NM_013544.2| |  |  |  |  |  |  |  |  |  | 0.02(0.04) |
| Arl4a | gi|87252732|ref|NM_007487.3| | 0.3(0.0006) |  |  |  |  |  |  |  |  |  | Hdc | gi|142385676|ref|NM_008230.5| |  |  |  |  |  |  |  |  |  | 8(0.04) |
| Prss29 | gi|31981507|ref|NM_053260.2| | 16.7(0.0006) | 195(2E-21)  302(3.2E-15) |  |  | 19(4E-35)  0.01(3E-14) |  | 11.7(1.3E-05)  7.1(2.5E-05) | 107(3.4E-30)  66(2.7E-38) | 65(2.2E-54)  3.5(0.02) | 9.1(0.0006) | LOC674994 | gi|94364096|ref|XM_982827.1| |  |  |  |  |  |  |  |  |  | 0.13(0.04) |
| Fosl2 | gi|40789304|ref|NM_008037.3| | 0.27(0.0006) |  |  |  |  |  |  |  |  |  | Il15 | gi|6680406|ref|NM_008357.1| |  |  |  |  |  |  | 4.6(0.01)  8.3(3.2E-07) | 9.8(0.0001)  18(2.1E-32) | 17.5(1.6E-44)  16.4(5E-22) |  |
|  | gi|94374443|ref|XM_909130.2| | 0.27(0.0007) |  |  |  |  |  |  |  |  |  | Hsd3b6 | gi|31980731|ref|NM_013821.2| |  |  |  |  |  |  |  | 13.1(0.003)  22(1E-13) | 26.5(1.8E-17)  14.9(2E-15) |  |
| Cldn5 | gi|142348145|ref|NM_013805.3| | 0.15(0.0007) |  |  |  |  |  |  |  |  |  | Saa3 | gi|118130197|ref|NM_011315.3| |  |  |  |  |  |  | 9.9(0.04)  35.3(3.6E-14) |  | 48.6(2.5E-13)  9.5(0.004) |  |
| Gja1 | gi|21687178|ref|NM_010288.2| | 0.33(0.0008) | 0.24(0.05)  0.12(0.04) | 0.15(0.01)  0.13(2.3E-05) |  |  |  |  |  |  |  | Zygin1 | gi|142349734|ref|NM_183171.2| |  |  |  |  |  |  | 95.6(1.9E-11)  30(7.6E-05) | 64(8.8E-08)  20.3(0.0004) | 56(6.2^E^-13)  14(0.002) |  |
| Gimap4 | gi|56549086|ref|NM_174990.3| | 0.12(0.001) |  |  |  |  |  |  |  |  |  | Gja4 | gi|34328122|ref|NM_008120.2| |  |  |  |  |  |  | 12.9(0.0001) |  | 11.9(7.7E-11)  10.8(1.6E-11) |  |
| Anxa1 | gi|124517662|ref|NM_010730.2| | 0.35(0.001) |  |  |  |  |  |  |  |  |  | Prr9 | gi|125991229|ref|NM_175424.3| |  |  |  |  |  |  | 13(2E-07)  5.3(0.001) | 20.7(0.0002)  13.8(6.7E-07) | 23.3(6E-10)  15.5(1.2E-10) |  |
| Jam2 | gi|142352774|ref|NM_023844.3| | 0.19(0.001) |  |  |  |  |  |  |  |  |  | Tmem45a | gi|142373885|ref|NM_019631.3| |  |  |  |  |  |  |  | 5.7(0.01)  9(1E-15) | 6.4(5.5E-09)  7.8(2.6E-17) |  |
| Gcnt3 | gi|124430728|ref|NM_028087.2| | 0.2(0.001) |  |  |  |  |  |  |  |  |  | Inhba | gi|6680450|ref|NM_008380.1| |  |  |  |  |  |  |  |  | 6.3(1E-06)  4.3(1.5E-05) |  |
| Kazald1 | gi|142353193|ref|NM_178929.3| | 0.18(0.001) |  | 0.13(0.03)  0.07(9.9E-05) |  |  |  |  |  |  |  | Scand1 | gi|142384255|ref|NM_020255.2| |  |  |  | 0.05(2.9E-07)  0.05(2E-41) |  |  |  | 0.01(0.0001)  0.09(0.0005) | 0.04(1.6E-06)  0.06(6E-10) |  |
| Tlr4 | gi|118130391|ref|NM_021297.2| | 0.14((0.002) |  |  |  |  |  |  |  |  |  | Adh1 | gi|21735490|ref|NM_007409.2| |  |  |  |  |  |  | 11.8(0.001)  10.4(0.0004) | 11.8(0.02)  10.6(0.0003) | 12.1(0.0001)  14.2(1.6E-11) |  |
|  | gi|149251850|ref|XR_034037.1| | 0.07((0.002) |  |  |  |  |  |  |  |  |  | Cda | gi|58037288|ref|NM_028176.1| |  |  |  |  |  |  | 5.4(0.01)  6.3(0.0006) |  | 5.3(0.0003)  3.5(0.003) |  |
| Tmem204 | gi|47564089|ref|NM_001001183.1| | 0.12(0.002) |  |  |  |  |  |  | 11.1(0.007)  6.6(0.004) |  |  | Prl6a1 | gi|6755167|ref|NM_011166.1| |  |  |  |  |  |  |  |  | 4.3(0.0003)  3.4(0.0009) |  |
| Ahnak2 | gi|85702000|ref|NM_001033476.1| | 0.16(0.002) |  |  |  |  |  |  |  |  |  | Serpina1b | gi|93102380|ref|NM_009244.4| |  |  |  |  |  |  | 22.6(0.0004)  18.4(0.0003) | 53(0.0009)  43.7(3.5E-06) | 33.7(0.0006)  17.1(0.01) |  |
|  | gi|149268975|ref|XR_033381.1| | 0.27(0.002) |  |  |  |  |  |  |  |  |  | Sdcbp2 | gi|21704053|ref|NM_145535.1| |  |  |  |  |  |  |  | 23.6(0.01)  16.1(0.003) | 20.7(0.0006)  13.4(0.003) |  |
|  | gi|149260864|ref|XM_001478148.1| | 0.25(0.002) |  |  |  |  |  |  |  |  |  | Lmcd1 | gi|21450156|ref|NM_144799.1| |  |  |  |  |  |  |  |  | 5.3(0.001)  3.8(0.003) |  |
| Rgs5 | gi|141801698|ref|NM_009063.3| | 0.31(0.002) |  |  |  |  |  |  |  |  |  | Tpsab1 | gi|31981623|ref|NM_031187.2| |  |  |  |  |  |  |  |  | 0.15(0.001)  0.17(9.5E-06) |  |
| Efemp1 | gi|22122482|ref|NM_146015.1| | 0.11(0.002) |  |  |  |  |  |  |  |  |  | Prl2c5 | gi|49258207|ref|NM_181852.1| |  |  |  |  |  |  |  | 0.16(0.005)  0.22(2.4E-06) | 0.27(0.002)  0.37(0.01) |  |
| Them4 | gi|110626049|ref|NM_029431.1| | 0.32(0.003) |  |  |  |  |  |  |  |  |  | Tnnc1 | gi|133892226|ref|NM_009393.2| |  |  |  |  |  |  |  |  | 3.6(0.003)  2.8(0.006) |  |
| Tspan2 | gi|70608156|ref|NM_027533.2| | 0.21(0.003) |  |  |  |  |  |  |  |  |  | H2-Ab1 | gi|144227216|ref|NM_207105.2| |  |  |  |  |  |  |  |  | 8.2(0.003)  4.7(0.04) |  |
| Vsig2 | gi|114145510|ref|NM_020518.2| | 0.14(0.004) |  |  |  |  |  |  |  |  |  | Adamts1 | gi|47087139|ref|NM_009621.3| |  |  |  |  |  |  |  |  | 3.6(0.004)  2.7(0.01) |  |
| Parp3 | gi|25014094|ref|NM_145619.2| | 0.2(0.004) |  |  |  |  |  |  |  |  |  | Calm4 | gi|142351881|ref|NM_020036.3| |  |  |  |  |  |  | 32.1(0.01)  34.7(0.002) |  | 25.2(0.004)  15.8(0.03) |  |
| Abcd1 | gi|6671496|ref|NM_007435.1| | 0.13(0.004) |  |  |  |  |  |  |  |  |  | Ifit3 | gi|6754287|ref|NM_010501.1| |  |  |  |  |  |  |  |  | 8.8(0.004)  7(0.002) |  |
| Arrdc4 | gi|112363103|ref|NM_001042592.2| | 0.25(0.004) |  |  |  |  |  |  |  |  |  | Ear6 | gi|32891936|ref|NM_053111.2| |  |  |  |  |  |  | 0.19(0.01)  0.18(0.004) |  | 0.23(0.005)  0.12(4.5E-10) |  |
|  | gi|142351874|ref|NM_145450.3| | 0.23(0.004) |  |  |  |  |  |  |  |  |  | Prl7d1 | gi|6755101|ref|NM_011120.1| |  |  |  |  |  |  |  | 0.07(0.01)  0.12(0.001) | 0.14(0.006)  0.18(0.001) |  |
|  | gi|149263928|ref|XM_001475956.1| | 0.28(0.005) |  |  |  |  |  |  |  |  |  | Cxcl13 | gi|118130712|ref|NM_018866.2| |  |  |  |  |  |  |  |  | 24(0.006)  29(6.9E-05) |  |
| Tacc1 | gi|110681725|ref|NM_199323.2| | 0.37(0.005) |  |  |  |  |  |  |  |  |  | Akr1c18 | gi|141802036|ref|NM_134066.2| |  |  |  |  |  |  |  |  | 0.07(0.009)  0.13(0.02) |  |
| Mib2 | gi|110347530|ref|NM_145124.2| | 5(0.005) |  |  |  |  |  |  |  |  | 0.06(3.4E-06) | Scara5 | gi|22296588|ref|NM_028903.1| |  |  |  |  |  |  |  |  | 5.5(0.009)  4.9(0.001) |  |
| Arl4a | gi|87252728|ref|NM_001039515.1| | 0.33(0.005) |  |  |  |  |  |  |  |  |  | Abca1 | gi|90568037|ref|NM_013454.3| |  |  |  |  |  |  |  |  | 3.5(0.01)  2.8(0.02) |  |
| Nmnat2 | gi|57863769|ref|NM_175460.3| | 0.27((0.006) |  |  |  |  |  |  |  |  |  | Caecam15 | gi|149256512|ref|XM_145393.5| |  |  |  |  |  |  |  |  | 3.2(0.01)  4.4(2.6E-08) |  |
| Pmaip1 | gi|118130467|ref|NM_021451.2| | 0.32((0.006) |  |  |  |  |  |  |  |  |  | LOC667370 | gi|94405885|ref|XM_001000862.1| |  |  |  |  |  |  |  | 21.2(0.006)  10.3(0.01) | 11.7(0.01)  10.8(0.002) |  |
|  | gi|118129953|ref|NM_172777.2| | 0.09(0.006) |  |  |  |  |  |  |  |  |  | Htra3 | gi|110815868|ref|NM_030127.2| |  |  |  |  |  |  |  | 13.4(0.006)  10.9(6.8E-05) | 7.7(0.01)  7.1(0.0009) |  |
| Lims2 | gi|142347134|ref|NM_144862.2| | 0.08(0.006) |  |  |  |  |  |  |  |  |  | Caecam15 | gi|149256851|ref|XM_914594.3| |  |  |  |  |  |  |  |  | 3.1(0.01)  4.4(3.9E-08) |  |
| Gsdmds1 | gi|142348546|ref|NM_026960.2| | 0.18(0.006) |  |  |  |  |  |  |  |  |  | Cidea | gi|6680943|ref|NM_007702.1| |  |  |  |  |  |  |  |  | 5.2(0.01)  4(0.01) |  |
| St8sia4 | gi|6677968|ref|NM_009183.1| | 0.18(0.007) |  |  |  |  |  |  |  |  |  | Tgtp2 | gi|149261867|ref|XR_031267.1| |  |  |  |  |  |  |  |  | 7.5(0.01)  6.5(0.004) |  |
| LOC100047300 | gi|149260446|ref|XM_001478776.1| | 3.47(0.007) | 7.3(9.9E-05)  6.1(0.04) | 7.9(0.0002)  19.6(1E-24) |  |  |  |  |  |  |  | Gdpd3 | gi|110431345|ref|NM_024228.2| |  |  |  |  |  |  | 0.12(0.001)  0.12(0.0003) |  | 0.2(0.02)  0.2(0.001) |  |
|  | gi|149263684|ref|XM_001477593.1| | 0.19(0.007) |  |  |  |  |  |  |  |  |  | Nceh1 | gi|142363800|ref|NM_178772.3| |  |  |  |  |  |  |  |  | 3.2(0.02)  2.5(0.03) |  |
|  | gi|149272419|ref|XM_001472704.1| | 0.28(0.007) |  | 0.18(0.04)  0.2(0.02) |  |  |  |  |  |  |  | Dio2 | gi|132566530|ref|NM_010050.2| |  |  |  |  |  |  |  | 9.6(0.007)  7(0.0001) | 5.2(0.02)  4.8(0.002) |  |
| Nrcam | gi|142377989|ref|NM_176930.3| | 0.24(0.007) |  |  |  |  |  |  |  |  |  | 0610012G03 | gi|149267321|ref|XR_035420.1| |  |  |  |  |  |  |  |  | 0.25(0.02)  0.26(0.002) |  |
| Lamb2 | gi|31982222|ref|NM_008483.2| | 0.33(0.007) |  |  |  |  |  |  |  |  |  | A230051G13 | gi|141802558|ref|NM_173785.4| |  |  |  |  |  |  |  |  | 0.24(0.02)  0.21(0.0002) |  |
| Bmp2 | gi|71896668|ref|NM_007553.2| | 0.34(0.008) |  |  |  |  |  |  |  |  |  | Gzmg | gi|70778833|ref|NM_010375.2| |  |  |  |  |  |  | 0.1(3E-06)  0.1(3E-07) |  | 0.28(0.03)  0.14(2.2E-09) |  |
|  | gi|149251752|ref|XM_001471642.1| | 0.14(0.008) |  |  |  |  |  |  |  |  |  | Irx2 | gi|47059033|ref|NM_010574.2| |  |  |  |  |  |  |  |  | 0.29(0.03)  0.28(0.001) |  |
| Dkk2 | gi|66955883|ref|NM_020265.3| | 0.25(0.008) |  | 0.12(0.009)  0.19(0.02) |  |  |  |  |  |  |  | LOC10004561 | gi|149265960|ref|XM_001474612.1| |  |  |  |  |  |  |  |  | 0.22(0.03)  0.24(0.005) |  |
| Ggtla1 | gi|141801950|ref|NM_011820.3| | 0.17(0.008) |  | 0.1(0.03)  0.11(0.009) |  |  |  |  |  |  |  | Tgtp | gi|31543860|ref|NM_011579.2| |  |  |  |  |  |  |  |  | 5.3(0.05)  7.4(3E-06) |  |
|  | gi|149269644|ref|XM_001474216.1| | 0.29(0.008) |  |  |  |  |  |  |  |  |  | Agr2 | gi|118130044|ref|NM_011783.2| |  |  |  |  |  |  | 381(1.5E-13)  240(3.8E-12) | 85(2.3E-08)  54.4(5.7E-09) |  |  |
| Hoxa11 | gi|118129999|ref|NM_010450.2| | 0.26(0.008) |  | 0.12(0.008)  0.09(7.7E-05) |  |  |  |  |  |  |  | Cwh43 | gi|31342193|ref|NM_181323.2| |  |  |  |  |  |  | 25.8(1.5E-12)  10.3(0.0009) | 32.8(3.5E-06)  13.3(0.0001) |  |  |
| Igsf11 | gi|40254204|ref|NM_170599.2| | 0.27(0.009) |  | 0.17(0.03)  0.13(0.001) |  |  |  |  |  |  |  | Actg2 | gi|6752951|ref|NM_009610.1| |  |  |  |  |  | 10.8(3.2E-05)  37.4(2.8E-29) | 15.1(2.3E-10)  19.7(3.1E-24) | 16.6(6E-06)  22(6.1E-33) |  |  |
|  | gi|149260757|ref|XM_001478007.1| | 0,26(0.009) |  |  |  |  |  |  |  |  |  | LOC100041932 | gi|149253162|ref|XM_001477842.1| |  | 11.5(1.5E-09)  6.7(0.02) |  |  | 5(7E-11)  0.21(0.02) |  | 13(2E-07)  5.2(0.001) | 21.7(4.5E-10)  8.9(6.5E-20) |  |  |
| Amotl1 | gi|124487106|ref|NM_001081395.1| | 0.38(0.01) |  |  |  |  |  |  |  |  |  | LOC100041156 | gi|149258996|ref|XM_001476722.1| |  | 10(4.8E-08)  6(0.04) |  |  | 4.8(1.6E-10)  0.23(0.03) |  | 12.7(4.6E-07)  5.2(0.001) | 25.2(2.4E-10)  10.5(8.7E-20) |  |  |
| Thy1 | gi|134152684|ref|NM_009382.3| | 0.14(0.01) |  |  |  |  |  |  |  |  |  | LOC100042156 | gi|149261633|ref|XR_033781.1| |  |  |  |  |  |  | 10.4(5.8E-07)  4.1(0.02) | 15.5(7E-09)  6.3(4.3E-09) |  |  |
| Ramp3 | gi|142384664|ref|NM_019511.2| | 0.18(0.01) |  |  |  |  |  |  |  |  |  | Myh11 | gi|7305294|ref|NM_013607.1| |  |  |  |  |  | 14.8(1.7E-06)  51.2(1.1E-30) | 10.3(1.7E-06)  13.8(2.6E-11) | 13.1(0.0001)  17.9(4.2E-16) |  |  |
| Angpt4 | gi|6753005|ref|NM_009641.1| | 0.09(0.01) |  |  |  |  |  |  |  |  |  | Dbpht2 | gi|115311549|ref|NM_198866.2| |  |  |  |  |  |  | 0.13(0.0001)  0.23(0.02) |  |  |  |
| Wt1 | gi|120444921|ref|NM_144783.2| | 0.28(0.01) |  |  |  |  |  |  |  |  |  | Cnn1 | gi|142347862|ref|NM_009922.3| |  |  |  |  |  | 8.3(0.001)  10.3(1.7E-12) | 8(0.0002)  8.9(1.5E-06) | 8(0.01)  8.9(2.3E-09) |  |  |
| Ugt1a6b | gi|47059132|ref|NM_201410.1| | 0.08(0.01) |  |  |  |  |  |  |  |  |  | Nupr1 | gi|9790144|ref|NM_019738.1| |  |  |  |  |  |  | 7.5(0.0003)  5.3(0.007) | 6.3(0.03)  4.5(0.004) |  |  |
|  | gi|149261778|ref|XM_001475752.1| | 0.2(0.01) |  |  |  |  |  |  |  |  |  | Gzmf | gi|116325973|ref|NM_010374.3| |  |  |  |  |  |  | 0.14(0.0004)  0.13(9.6E-06) |  |  |  |
| Lsamp | gi|118130292|ref|NM_175548.3| | 0.05(0.01) |  |  |  |  |  |  |  |  |  | H2-Eb1 | gi|114431227|ref|NM_010382.2| |  |  |  |  |  |  | 24.9(0.0006)  19.9(0.0003) | 14(0.03)  11.6(0.004) |  |  |
| Samd12 | gi|144446081|ref|NM_177225.3| | 0(0.01) |  |  |  |  |  |  |  |  |  | Col11a1 | gi|124487345|ref|NM_007729.2| |  |  |  |  |  |  | 9.4(0.001)  7.1(0.004) | 8(0.04)  6.2(0.004) |  |  |
| Cyp2j11 | gi|51921286|ref|NM_001004141.1| | 0.05(0.01) |  |  |  |  |  |  |  |  |  | Acta2 | gi|31982518|ref|NM_007392.2| |  |  |  |  |  | 7.2(0.01)  11.3(1.1E-23) | 6.7(0.001)  4.5(0.009) | 23.6(3.3E-12)  15.9(1.5E-20) |  |  |
| Sparcl1 | gi|31982799|ref|NM_010097.2| | 0.26(0.01) |  | 0.13(0.01)  0.09(7E-05) |  |  |  |  | 6.7(0.006)  3.8(0.003) |  |  | Pcp4 | gi|52694684|ref|NM_008791.2| |  |  |  |  |  | 13.6(0.002)  ??(1.1E-14) | 14.4(0.001)  17.8(3.6E-06) | 41.7(0.0009)  52.4(1E-08) |  |  |
| Hdac9 | gi|70778860|ref|NM_024124.2| | 0.14(0.01) |  |  |  |  |  |  |  |  |  | Mylk | gi|126157498|ref|NM_139300.3| |  |  |  |  |  |  | 5.6(0.002)  8.2(1E-06) | 8.3(0.001)  12.3(7E-16) |  |  |
|  | gi|149266741|ref|XM_001478533.1| | 0(0.01) |  |  |  |  |  |  |  |  |  | Itga8 | gi|118131074|ref|NM_001001309.2| |  |  |  |  |  |  | 5.8(0.005)  4.9(0.01) | 9.5(0.005)  8.3(7.8E-07) |  |  |
| Fgf5 | gi|141802811|ref|NM_010203.3| | 4.2(0.01) |  | 6.9(0.01)  12.8(3E-08) |  |  |  |  |  |  |  | Tagin | gi|142362320|ref|NM_011526.3| |  |  |  |  |  | 5(0.04)  18.1(1.2E-25) | 5(0.005)  5.9(0.0003) | 13.9(3.2E-06)  16.3(4.2E-22) |  |  |
| LOC100047285 | gi|149269087|ref|XM_001477507.1| | 9(0.01) | 126(3.5E-22)  200(1.8E-15) |  |  | 19.6(2E-36)  0.01(1.4E-15) |  | 12.3(1.8E-05)  7.6(7.6E-06) | 116(1E-30)  73(8E-66) | 72(2.5E-56)  3.7(0.007) | 9.4(0.0003) | Zfp367 | gi|141802889|ref|NM_175494.3| |  |  |  |  |  |  | 0.18(0.007)  0.16(0.0007) |  |  |  |
| C1qtnf1 | gi|133892163|ref|NM_019959.2| | 0.26(0.01) |  |  |  |  |  |  |  |  |  | Lmod1 | gi|134948540|ref|NM_053106.2| |  |  |  |  |  |  | 16.5(0.009)  23.9(8.8E-06) |  |  |  |
| Atp8b1 | gi|95007005|ref|NM_001001488.3| | 0.21(0.02) |  |  |  |  |  |  |  |  |  | Gzme | gi|116325996|ref|NM_010373.3| |  |  |  |  |  |  | 0.15(0.01)  0.18(0.02) |  |  |  |
| Ano10 | gi|141802585|ref|NM_133979.2| | 0.35(0.02) |  |  |  |  |  |  |  |  |  | Gzmd | gi|6754105|ref|NM_010372.1| |  |  |  |  |  |  | 0.21(0.02)  0.2(0.008) |  |  |  |
| Tnfaip2 | gi|6678374|ref|NM_009396.1| | 0.33(0.02) |  |  |  |  |  |  |  |  |  | Synpo2 | gi|94369843|ref|XM_619958.3| |  |  |  |  |  |  | 6.8(0.03)  7(0.006) |  |  |  |
| Cyp4v3 | gi|118129905|ref|NM_133969.2| | 0.1(0.02) |  |  |  |  |  |  |  |  |  | LOC100044263 | gi|149257836|ref|XM_001471609.1| |  | 0.03(3E-06)  0.05(0.006) | 0.01(4.7E-06)  0.004(2.4E-10) | 0.03(8.4E-06)  0.009(7.6E-08) |  |  |  |  |  |  |
| Cst9 | gi|6753545|ref|NM_009979.1| | 0(0.02) |  |  |  |  |  |  |  |  |  | Arg1 | gi|31982504|ref|NM_007482.2| |  | 0.05(2.9E-05)  0.03(0.002) | 0.1(0.01)  0.07(6.8E-05) | 0.15(0.02)  0.1(0.0005) |  |  |  |  |  |  |
| Dhrs3 | gi|142370009|ref|NM_011303.3| | 0.38(0.02) |  |  |  |  |  |  | 5.8(0.01)  2.7(0.04) |  |  | Hbb-bh1 | gi|31982298|ref|NM_008219.2| |  | 0.05(0.0005)  0.04(0.01) | 0.04(0.0009)  0(9.8E-09) | 0.08(0.02)  0(7E-06) |  |  |  |  |  |  |
|  | gi|149249534|ref|XR_034465.1| | 0.3(0.02) |  |  |  |  |  |  |  |  |  | Prrx2 | gi|6677840|ref|NM_009116.1| |  | 0.14(0.02)  0.08(0.02) | 0.14(0.04)  0.08(0.0001) |  |  |  |  |  |  |  |
| Bcmo1 | gi|11993945|ref|NM_021486.2| | 0.33(0.02) |  |  |  |  |  |  |  |  |  | LOC100046051 | gi|149251661|ref|XM_001475822.1| |  |  |  | 0.1(8.4E-06)  0.13(6.4E-05) |  |  |  |  |  |  |
| B3galnt1 | gi|141803426|ref|NM_020026.3| | 0.36(0.02) |  |  |  |  |  |  |  |  |  | Apol6 | gi|149266960|ref|XM_484487.5| |  |  |  | 0.12(0.008)  0.09(0.0002) |  |  |  |  |  |  |
| Ugt1a1 | gi|47059134|ref|NM_201645.1| | 0.06(0.02) |  |  |  |  |  |  |  |  |  | Zfp503 | gi|42734490|ref|NM_145459.3| |  |  | 0.13(0.0004)  0.06(2.3E-10) | 0.23(0.01)  0.11(7E-13) |  |  |  |  |  |  |
|  | gi|142363148|ref|NM_027519.2| | 0.35(0.02) |  | 0.17(0.01)  0.19(0.007) |  |  |  |  |  |  |  | Aldh1a2 | gi|144227213|ref|NM_009022.3| |  |  | 0.16(0.002)  0.04(1.9E-33) | 0.25(0.01)  0.05(2.7E-41) |  |  |  | 8.2(0.0004)  3.7(0.0007) |  |  |
| Gpr64 | gi|119943130|ref|NM_001079857.1| | 0.09(0.02) |  |  |  |  |  |  |  |  |  | Cdx1 | gi|46559385|ref|NM_009880.2| |  |  | 0.08(0.01)  0.04(5E-05) | 0.11(0.01)  0.06(0.0004) |  |  |  |  |  |  |
| Med30 | gi|141801965|ref|NM_027212.2| |  |  |  | 0.18(0.02)  0.19(0.02) |  |  |  |  |  |  | Ggh | gi|100815971|ref|NM_010281.2| |  |  |  |  |  |  |  | 5.8(0.02)  5.1(5.7E-06) |  |  |
| Cfh | gi|109627651|ref|NM_009888.3| |  |  |  |  |  |  |  | 7.2(0.003)  3.3(0.02) |  |  | CD74 | gi|110624769|ref|NM_001042605.1| |  |  |  |  |  |  |  | 8.2(0.02)  4.9(0.02) |  |  |
| CD74 | gi|110624778|ref|NM_010545.3| |  |  |  |  |  |  |  | 7.7(0.03)  4.4(0.03) |  |  | Ramp1 | gi|110625604|ref|NM_016894.2| |  |  |  |  |  |  |  | 10(0.02)  6.9(0.004) |  |  |
| Ccdc80 | gi|110625751|ref|NM_026439.2| |  |  |  |  |  |  |  | 8.2(0.02)  5.8(0.003) |  |  | Robo2 | gi|110815825|ref|NM_175549.4| |  |  |  |  |  |  |  | 9.7(0.0009)  4.4(0.006) |  |  |
| Col1a2 | gi|111120328|ref|NM_007743.2| |  |  |  |  |  |  |  | 7.6(0.0009)  3.7(0.0001) |  |  | Prl2C1 | gi|113865900|ref|NM_001045532.1| |  |  |  |  |  |  |  | 0.19(0.02)  0.2(3.5E-07) |  |  |
| Cdh11 | gi|114687887|ref|NM_009866.4| |  |  |  |  |  |  |  | 5.6(0.02)  3.1(0.02) |  |  | Col6a1 | gi|118129824|ref|NM_009933.2| |  |  |  |  |  |  |  | 8(0.0008)  3.7(0.002) |  |  |
| Mmp23 | gi|118129979|ref|NM_011985.2| |  |  |  |  |  |  |  | 16.8(3.8E-08)  5.5(0.01) |  |  | Cwh43 | gi|118130159|ref|NM_145560.2| |  |  |  |  |  |  |  | 21(0.02)  11.5(0.03) |  |  |
| Lgals3bp | gi|118130236|ref|NM_011150.2| |  |  |  |  |  |  |  | 14.3(0.0005)  6.8(0.002) |  |  | Col1a1 | gi|118131144|ref|NM_007742.3| |  |  |  |  |  |  |  | 5.4(0.01)  3.3(0.001) |  |  |
| Tcf23 | gi|118131202|ref|NM_053085.2| |  |  |  |  |  |  |  | 12.7(0.007)  7.3(0.005) |  |  | Sema5a | gi|119226252|ref|NM_009154.2| |  |  |  |  |  |  |  | 5.5(0.03)  3.4(0.02) |  |  |
| Itih5 | gi|124286821|ref|NM_172471.2| |  |  |  |  |  |  |  | 9.2(0.0008)  4.3(0.004) |  |  | Axl | gi|133892528|ref|NM_009465.3| |  |  |  |  |  |  |  | 7.8(0.02)  5.8(0.002) |  |  |
| C1qb | gi|133893071|ref|NM_009777.2| |  |  |  |  |  |  |  | 16.6(0.004)  11.4(0.0005) |  |  | Prl2C3 | gi|13654299|ref|NM_011118.1| |  |  |  |  |  |  |  | 0.2(0.04)  0.26(0.0001) |  |  |
| Wfdc2 | gi|141802067|ref|NM_026323.2| |  |  |  |  |  |  |  | 11.5(1.1E-05)  4(0.002) |  |  | Gem | gi|141802514|ref|NM_010276.2| |  |  |  |  |  |  |  | 7.8(0.04)  5.4(0.  01) |  |  |
| Tgfbi | gi|141803530|ref|NM_009369.2| |  |  |  |  |  |  |  | 10.2(0.002)  4.9(0.01) |  |  | Spaca7 | gi|142361666|ref|NM_024279.2| |  |  |  |  |  |  |  | 30.6(0.02)  32.3(0.0004) |  |  |
| Chodl | gi|142365147|ref|NM_139134.3| |  |  |  |  |  |  |  | 10.1(0.005)  6.4(0.001) |  |  | Prl5a1 | gi|142367178|ref|NM_023746.4| |  |  |  |  |  |  |  | 0.16(0.004)  0.4(0.05) |  |  |
| Ptgds2 | gi|142382100|ref|NM_019455.3| |  |  |  |  |  |  |  | 11.3(0.01)  11.9(1.8E-06) |  |  | 5730469M10Rik | gi|142387361|ref|NM_027464.2| |  |  |  |  |  |  |  | 5.2(0.02)  3(0.004) |  |  |
| Myl9 | gi|149250223|ref|XM_485171.5| |  |  |  |  |  |  |  | 8.8(0.008)  6.9(5.3E-05) |  |  | Myl9 | gi|149250365|ref|XM_977718.2| |  |  |  |  |  |  |  | 9.8(0.004)  8.3(7.8E-07) |  |  |
| Ggh | gi|149252055|ref|XM_989632.2| |  |  |  |  |  |  |  | 5(0.05)  4.6(6.1E-05) |  |  | LOC100046795 | gi|149258827|ref|XM_001476826.1| |  |  |  |  | 3(0.004)  0.23(0.03) |  |  | 7.8(0.003)  4.4(0.001) |  |  |
| Gm7040 | gi|149263882|ref|XM_001473551.1| |  |  |  |  |  |  |  | 0.19(0.01)  0.3(0.003) |  |  | Gm7040 | gi|149263978|ref|XM_904245.3| |  |  |  |  |  |  |  | 0.19(0.02)  0.3(0.001) |  |  |
| Sult1a1 | gi|19526821|ref|NM_133670.1| |  |  |  |  |  |  |  | 13.3(0.0009)  7(0.001) |  |  | 8430408G22 | gi|22122424|ref|NM_145980.1| |  |  |  |  |  |  |  | 14.2(2E-05)  7.1(1.7E-05) |  |  |
| Col6a2 | gi|22203746|ref|NM_146007.1| |  |  |  |  |  |  |  | 15(1.6E-07)  5.8(6E-06) |  |  | Bgn | gi|24475819|ref|NM_007542.3| |  |  |  |  |  |  |  | 11.1(2.3E-05)  4.8(8.4E-05) |  |  |
| Vtcn1 | gi|31341500|ref|NM_178594.2| |  |  |  |  |  |  |  | 24.4(0.0008)  11.6(0.002) |  |  | RNAse1 | gi|31981519|ref|NM_011271.2| |  |  |  |  |  |  |  | 42(0.0001)  29.5(2.2E-06) |  |  |
| PamR1 | gi|31982079|ref|NM_173749.3| |  |  |  |  |  |  |  | 34.3(9.7E-06)  17(2.1E-05) |  |  | Mmp14 | gi|31982190|ref|NM_008608.2| |  |  |  |  |  |  |  | 5.6(0.01)  3.7(0.0009) |  |  |
| Ccdc109b | gi|31982669|ref|NM_025779.2| |  |  |  |  |  |  |  | 25.8(0.04)  17.4(0.03) |  |  | Col3a1 | gi|33859525|ref|NM_009930.1| |  |  |  |  |  |  |  | 6.5(0.003)  2.9(0.01) |  |  |
| Eaf2 | gi|40254104|ref|NM_134111.2| |  |  |  |  |  |  |  | 6.7(0.009)  3.4(0.02) |  |  | Wnt5a | gi|46909566|ref|NM_009524.2| |  |  |  |  |  |  |  | 6.2(0.02)  3.4(0.02) |  |  |
| Mmp2 | gi|47271505|ref|NM_008610.2| |  |  |  |  |  |  |  | 8.6(0.0009)  4.2(0.002) |  |  | sftpd | gi|6677920|ref|NM_009160.1| |  |  |  |  |  |  |  | 17.6(8E-05)  9.7(1.5E-05) |  |  |
| Fxyd3 | gi|6678813|ref|NM_008557.1| |  |  |  |  |  |  |  | 9.5(0.0008)  6.1(8.1E-06) |  |  | Rhoj | gi|70980541|ref|NM_023275.2| |  |  |  |  |  |  |  | 7(0.07)  4.5(0.0007) |  |  |
| Adamts15 | gi|71725382|ref|NM_001024139.1| |  |  |  |  |  |  |  | 8.4(0.01)  5.5(0.002) |  |  | Dnm3os | gi|84872167|ref|NR_002870.1| |  |  |  |  |  | 5.3(0.03)  12.4(1.3E-14) |  | 8.2(0.001)  4.6(0.0004) |  |  |
| Serpina1d | gi|93102381|ref|NM_009246.3| |  |  |  |  |  |  |  | ??(2.2E-06)  ??(0.008) |  |  | Serpina1a | gi|93102382|ref|NM_009243.3| |  |  |  |  |  |  |  | ??(0.0005)  ??(0.0002) |  |  |
| Tmem213 | gi|94377213|ref|XM_914650.2| |  |  |  |  |  |  |  | 19.1(0.001)  7.8(0.01) |  |  | Tmem213 | gi|94377270|ref|XM_885435.2| |  |  |  |  |  |  |  | 41.4(6.7E-05)  19(0.0002) |  |  |
| Hoxa1 | gi|144922691|ref|NM_010449.3| |  |  | 0.16(0.009)  0.08(2.3E-08) |  |  |  |  |  |  |  | EG620395 | gi|149267297|ref|XM_884793.3| |  |  | 13.2(0.01)  38.5(9.8E-14) |  |  |  |  |  |  |  |
| Pga5 | gi|142378195|ref|NM_021453.3| |  |  | 6(0.02)  7.3(0.0003) |  |  |  |  |  |  |  | Sdpr | gi|20270266|ref|NM_138741.1| |  |  | 0.12(0.02)  0.12(0.002) |  |  |  |  |  |  |  |
| Cyp2b10 | gi|118129803|ref|NM_009999.3| |  |  | 0.05(0.02)  0.02(0.0003) |  |  |  |  |  |  |  | EG620395 | gi|149267718|ref|XM_906462.3| |  |  | 10.6(0.02)  30.7(9.8E-14) |  |  |  |  |  |  |  |
| Hapln1 | gi|46048400|ref|NM_013500.3| |  |  | 0.18(0.02)  0.15(0.0004) |  |  |  |  |  |  |  | Slc22a5 | gi|31981489|ref|NM_011396.2| |  |  | 0.15(0.02)  0.17(0.007) |  |  |  |  |  |  |  |
| Ndufaf1 | gi|141803171|ref|NM_027175.3| |  |  | 4.4(0.03)  3.8(0.02) |  |  |  |  |  |  |  | Cer1 | gi|6753409|ref|NM_009887.1| |  |  | 4.4(0.03)  7(1.1E-05) |  |  |  |  |  |  |  |
| Klk1 | gi|142357330|ref|NM_010639.6| |  |  |  |  | 62(9.1E-15)  0(0.002) |  |  |  |  |  | Sult1d1 | gi|42734448|ref|NM_016771.2| |  |  |  |  | 27.7(1E-17)  0.07(0.0004) |  |  |  |  |  |
